# Statistical optimization of crude oil bioremediation using *Streptomyces aurantiogriseus* isolated from Egypt’s Western Desert

**DOI:** 10.1007/s10532-025-10154-0

**Published:** 2025-07-25

**Authors:** Sahar Y. Ibrahim, Eman A. Abdelhamid, Ali M. El-Hagrassi, Noha M. Kamal

**Affiliations:** 1 Botany Department, Faculty of Women for Arts, Science, and Education, Ain Shams University, Cairo, 11757 Egypt; 2 Department of Phytochemistry and Plant Systematics, Pharmaceutical Industries Research Institute, National Research Centre, 33 EL Buhouth St., Dokki, Giza, 12622 Egypt

**Keywords:** Pollution, *Streptomyces*, GC–MS, Identification, Hydrocarbons, Bioaugmentation, Biodegradation

## Abstract

One of the most common soil pollutants on a global scale is fuel, which is fundamental for daily activities. Biodegradation has been regarded as an ideal remediation technique for hydrocarbon pollution. We investigated the potential of 28 *Streptomyces* species inhabiting different hydrocarbon-polluted soils for the biodegradation of petroleum. The tested isolates were cultured on mineral salts broth containing 2% crude oil as the sole carbon source. Gravimetric analysis of residual crude oil was performed, and the samples that showed the highest percentage of biodegradation were also analyzed via gas chromatography. Among the isolated actinobacteria, *Streptomyces aurantiogriseus* strain NORA7 (EMCC 28565) stood out for its ability to degrade crude oil (66.28 ± 6.25%). Gas chromatography revealed that docosane, nonadecane, pentacosane, and 7-methylpentadecane were the major compounds detected in the residual treated crude oil. Plackett–Burman design (PB) was used to determine the critical factors impacting the biodegradation process. Response surface methodology (RSM) through Central Composite Design (CCD) was subsequently conducted, and the predicted optimum point of crude oil biodegradation was at 3% crude oil concentration, 0.15 g/L yeast extract, and 25 mm inoculum size. The experimental value after optimum conditions was 70% after 3 weeks, which was close to the predicted value. A pot experiment was performed to investigate the outcomes of ex situ soil bioremediation, and the results were consistent with those of the flask-scale biodegradation experiment with enhanced removal of crude oil (92%). The results revealed that the ability of *S. aurantiogriseus* NORA7 to biodegrade crude oil could significantly contribute to the eco-friendly recovery of oil-polluted ecosystems and reduce the long-term environmental impact of crude oil pollution.

**Graphical abstract:**

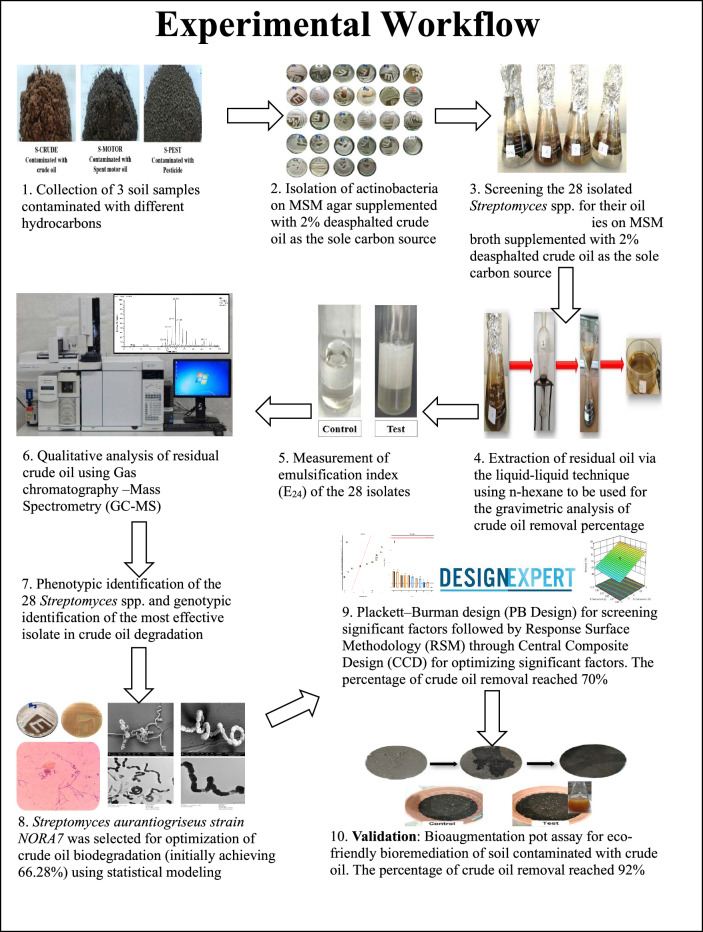

**Supplementary Information:**

The online version contains supplementary material available at 10.1007/s10532-025-10154-0.

## Introduction

Petroleum is another term for crude oil. Most crude oil comprises liquid hydrocarbons (HCs) that contain four or more carbon atoms. In petroleum, three primary categories of hydrocarbons exist: aromatics, cycloalkanes, and alkanes. (Soliman et al. 2021).

Crude oil, a fundamental global energy resource, has been instrumental in fueling cultural development and driving industrial advancement. Nevertheless, the extensive use of petroleum products has resulted in significant environmental issues: the pollution of ecosystems, water sources, and soils with crude oil and its byproducts. Regrettably, there are frequent reports of significant leakage and accidental spills of crude oil into the environment during various stages of petroleum exploration, production, refining, transportation, and storage (Baniasadi et al. 2009; Parikh et al. 2018; Robichaud et al. 2019). Crude oil spills, whether resulting from accidents during transportation or industrial operations, pose a significant threat to the environment, biodiversity, and human health (Klamerus-Iwan et al. 2015; Ali et al. 2020). Petroleum hydrocarbons are composed of complicated mixtures of nonaqueous and hydrophobic components, including n-alkanes, aromatic compounds, resins, and asphaltenes. The Environmental Protection Agency (EPA) classifies these compounds as “Priority Environmental Pollutants” because of their detrimental effects on human health and the environment (Ilori et al. 2005). Extensive changes in the local system may result from soil contamination with toxic hydrocarbons. The accumulation of these compounds in living organisms’ tissues may cause death or mutation. Some toxic hydrocarbons are classified as carcinogens and neurotoxic organic pollutants, which may disrupt the natural equilibrium between living species and their natural environment (Kuiper et al. 2004; Tugrul and Cansunar 2005; Olasehinde and Olaniran 2022). The urgent need for effective, sustainable, and environmentally friendly solutions to mitigate the impact of crude oil pollution has spurred extensive research into bioremediation strategies.

Bioremediation, a natural and eco-friendly approach, relies on the metabolic activities of microorganisms to degrade and transform hydrocarbon contaminants into less harmful substances. Many microorganisms can break down hydrocarbons, but actinobacteria have become very popular as bioremediation candidates because of their unique metabolic versatility and resilience in harsh environments.

Actinobacteria are among the most important groups of microorganisms. These filamentous bacteria are famous for their ability to prosper in variable ecological settings. They can produce unique, influential, and commercially useful bioactive metabolites, including enzymes capable of pollutant destruction and recycling. Therefore, they can be used in a variety of bioremediation processes, such as biotransformation and biodegradation (Devanshi et al. 2021). Some species belonging to *Streptomyces*, *Arthrobacter*, *Rhodococcus*, *Gordonia* and *Mycobacterium* are known hydrocarbon degraders (Parikh et al. 2018).

Bioremediation processes, which use native microorganisms to clean areas contaminated with hazardous organic residues, are constantly evolving (Vishwakarma et al. 2020). Recent advances in biotechnology, molecular biology, and environmental microbiology have expanded our understanding of actinobacterial diversity and their remarkable ability to metabolize complex hydrocarbons. This property has paved the way for harnessing the microbial ability of actinobacteria to address the complex challenges posed by crude oil degradation. In the realm of biotechnology, the bioremediation potential of actinobacteria has revealed fascinating prospects for a sustainable environment. Actinobacteria can be applied in in situ and ex situ bioremediation. In situ applications include bioaugmentation and biostimulation, whereas ex situ applications include slurry phase techniques (bioreactors) and solid phase techniques, such as biopiling, biofiltering and composting (Nano et al. 2003).

New strategies for crude oil bioremediation include several innovative approaches designed to increase the breakdown of hydrocarbons in contaminated environments. These include advanced biostimulation techniques, such as the use of nutrient-adsorbed clay flakes for the slow and sustained release of essential nutrients such as nitrogen and phosphorus, thereby maximizing the activity of indigenous microbial populations. Bioaugmentation remains a key strategy, focusing on the targeted introduction of highly effective hydrocarbon-degrading microbes, exemplified by *Pseudomonas aeruginosa*, to bolster existing microbial communities. Furthermore, the application of biosurfactants, which are naturally produced by microorganisms, is gaining prominence because of their ability to increase the bioavailability of oil by emulsifying hydrophobic compounds, making them more accessible for microbial degradation. In addition to bacterial methods, other biological strategies such as mycoremediation, which leverages fungi for pollutant degradation, and phytoremediation, which uses plants to absorb or degrade contaminants, are being explored as complementary approaches to conventional microbial degradation (Matilda and Samuel 2024). Other recent strategies for crude oil bioremediation focus on activating dormant or viable but nonculturable (VBNC) bacterial populations, often through the application of resuscitation-promoting factors (Rpfs). These factors stimulate the growth and activity of both indigenous and augmented microbial communities, increasing their crude oil degradation ability. Novel approaches also involve the simultaneous application of Rpfs with traditional biostimulation and bioaugmentation techniques, providing a more comprehensive remediation strategy. Furthermore, recent studies have explored the use of alternative resuscitation agents, such as supernatant Rpf (SRpf) and *Micrococcus luteus*, which have shown similar effectiveness to recombinant Rpf in enhancing total petroleum hydrocarbon removal (Tahmasbizadeh et al. 2025).

In this study, we explore the promising field of actinobacterial bioremediation to rejuvenate contaminated soil as an environmental scrubber by (a) exploring the diversity of actinobacteria in different contaminated soils, proceeding with phenotypic and genotypic approaches; (b) practical application in crude oil degradation via the batch culture technique; (c) gravimetric method and gas chromatography for crude oil analysis; and (d) ex situ bioremediation experiments using the most effective isolate.

## Materials and methods

### Chemicals used

The solvent required for crude oil analysis (n-hexane SupraSolv®) was GC-grade, and all other chemicals used for media preparation were analytical-grade. Deasphalted crude oil (DAO) was obtained kindly from Agiba Petroleum Company, Cairo, Egypt.

### Microbiological media

Mineral salts medium (MSM) at pH 7.0 used for actinobacterial growth and isolation. It consists of (g/L distilled water): NaCl, 1; KH_2_PO_4_, 1; Na_2_HPO_4_, 1; NH_4_NO_3_, 0.5; (NH_4_)_2_SO_4_, 0.5; MgSO_4_·7H_2_O, 0.2; CaCl_2_·2H_2_O, 0.02; FeCl_3_, 0.002 and MnSO_4_⋅2H_2_O, 0.002, and agar 2% (in the case of solid medium) (Khandelwal et al. 2022). Starch nitrate agar (SNA) was used for actinobacterial subculturing and preservation. Starch nitrate broth (SNB) with 50% glycerol was used and kept in freezer at −10 °C for isolate maintenance (Elumalai et al. 2021).

For actinobacterial phenotypic identification, Czapek’s medium (Atlas 2006) and ISP media (Shirling and Gottlieb 1966) were used. The ISP media used included yeast extract-malt extract agar (ISP2), oatmeal agar (ISP3), inorganic salt starch agar (ISP4), glycerol-asparagine agar (ISP5), peptone yeast extract iron medium (ISP6), tyrosine agar medium (ISP7), and carbon utilization agar medium (ISP9) (Pridham and Lyons 1961). Additionally, Bennet’s agar medium was used for testing sensitivity to streptomycin (Murray 2003).

### Microbial strains

Antimicrobial activity was performed for the identification of the tested actinobacteria. The strains tested for susceptibility to the tested actinobacteria were kindly obtained from the Botany Department, Faculty of Women for Arts, Science and Education, Ain Shams University. Two bacterial strains (*Bacillus subtilis* EMCCN 1152, *Klebsiella pneumonia* ATCC 2146), *Candida albicans* ATCC 10126, and two mold strains (*Aspergillus flavus* EMCCN 3033 and *Penicillium chrysogenum* EMCCN 10403) were used to test the antimicrobial activity of the studied actinobacteria.

### Soil analysis and actinobacteria isolation

Soil samples were collected from three sites polluted with complex hydrocarbons in Egypt. The description and locations of the collected polluted soil samples are provided in Supplementary Table S1. The soil samples were coded as S-CRUDE, S-MOTOR, or S-PEST, which correspond to soils contaminated with crude oil, motor oil, or pesticide, respectively. All the soil samples were collected at a depth of 15 cm, ground via a mortar and pestle, sieved through a 2-mm sieve, mixed well, air dried, placed in a sterile container, transferred directly to the laboratory under aseptic conditions, and stored at 4 °C for further investigations (Ferguson et al. 2020).

The physicochemical analysis of the soil samples, organic matter, pH, electrical conductivity (EC), elemental analysis, and hydrocarbon chromatographic analysis of the polluted soils was performed in the Central Lab Unit (CLU), Ain Shams University, Cairo, Egypt. The soil texture was determined via the United States Department of Agriculture (USDA) triangle by its fractions of sand, silt, and clay particles (Groenendyk et al. 2015). For elemental analysis, the soil samples were crushed with a porcelain mortar and pestle. Approximately 1 g of the sample was digested in 12 mL nitric acid (70% HNO_3_) in the CEM, MDS 2000 Microwave Digestion System following the microwave program. The digests were completed to 100 mL with deionized water and then diluted (1 mL in 100 mL deionized water). Metals were analyzed through flame atomic absorption spectroscopy (FAAS) employing a GBC scientific equipment model (Savant AA) with a metal hollow cathode lamp.

Hydrocarbons in S-CRUDE and S-MOTOR soils were also surveyed through gas chromatography analysis in the Central Lab Unit, ASU, Cairo, Egypt, via gas chromatography-mass spectrometry (GC–MS) (Agilent Technologies 7890B GC Systems combined with a 5977A mass selective detector), and S-PEST soil was analyzed in Nawah Scientific, Egypt, via GC–MS (Thermo Scientific™ TRACE™ 1310 GC system). The soil sample was dissolved in dichloromethane; then a capillary column was applied (HP-5MS capillary; 30.0 m × 0.25 mm ID × 0.25 μm film). The carrier gas was helium at a pressure of 7.6 psi with a 1 μl injection. The sample was analyzed with the column held initially for 3 min at 50 °C after injection, and then the temperature was increased to 300 °C with a 20 °C/min heating ramp, with an 8.0 min hold. Injection was carried out in split mode at a ratio of 1:1 at 300 °C. The MS scan range was (m/z): 50–550 atomic mass units (AMU) under electron impact (EI) ionization (70 eV) and a solvent delay of 4 min.

For actinobacteria isolation, 1 g of air-dried sample was mixed with 1 g of calcium carbonate. This mixture was incubated for 5 days at 55 °C in a sterile empty Petri dish to selectively suppress most bacteria and fungi while promoting the growth of actinobacteria, thereby yielding the highest relative count of actinobacteria (El-Nakeeb et al. 1963). Subsequently, serial dilutions (10^–3^, 10^–4^, 10^–5^) of these soil samples were prepared. An aliquot of each dilution was then inoculated onto MSM agar supplemented with 2% crude oil. The plates were incubated at 30 ± 2 °C for 5–7 days. After incubation, actinobacteria colonies were picked and subcultured several times to obtain pure cultures, when needed (Khandelwal et al. 2022). Young cultures were preserved by placing SNA plates at 4 °C for 2–3 weeks and were subsequently used for further investigations. A single colony of each isolate was also inoculated in a flask containing SNB and incubated at 30 ± 2 °C for 5–7 days. Then, the obtained culture was mixed gently with an equal volume of sterile glycerol (50%) in a 1.5 mL cryovial which was transferred immediately to a −70 °C freezer to prevent ice crystal formation and maintain viability for long-term storage. To recover actinobacteria from glycerol stock, a sterile loop was used to scrape a small amount of the frozen stock (without thawing the entire tube), which was subsequently streaked onto SNA plate which were incubated as described previously (Sáhó et al. 2024).

### Crude oil biodegradation assay

To survey the oil biodegradation capability of the isolated actinobacteria, a 10 mm ISP2 agar plug of 7-day-old isolate culture was inoculated in 100 mL of MSM broth supplemented with 2% deasphalted crude oil sterilized by autoclaving. The inoculated media were incubated at 30 °C in a Thermo Scientific MaxQ 4450 shaking incubator at 120 rpm for 1 week. The residual oil was extracted via the liquid–liquid technique. The organic solvent n-hexane was selected for residual crude oil extraction according to the modified protocol of Márquez-Rocha, Hernández-Rodrí, and Lamela (Márquez-Rocha et al. 2001). A volume of 50 mL of n-hexane was added to each culture broth and the mixture was then transferred to a separating funnel and stirred carefully. After the aqueous phase was removed, the organic phase was filtered through 10 g anhydrous sodium sulfate for moisture elimination, and the filtrate was collected in preweighed flasks. The extract was left overnight for organic solvent evaporation until drying, and the residual crude oil weight was deduced, followed by gravimetric and GC–MS analyses. The negative control was actinobacteria-free MSM broth with 2% DAO. For gravimetric analysis, the biodegradation percentage was calculated as shown in Eqs. (1) and (2) (Soumeya et al. 2022).

1 $$\text{Weight ofdegraded crude oil }=\text{ Weight of crude oil added in the media}-\text{ Weight of residual crude oil}$$

2 $$\text{The percentage of degradation }=\text{ Weight of degraded crude oil }\div \text{ Weight of crude oil added in the media }\times 100$$

### Emulsification index assay

The capacity of the actinobacteria to produce biosurfactants was also screened for all the isolates in terms of the emulsification index (E_**24**_); it was assessed according to Ilori et al. (2005) and Soumeya et al. (2022). To evaluate E_**24**_, the isolate cell-free supernatant (CFS) was obtained by filtration of the isolate broth culture, and the filtrate was centrifuged at 10,000 rpm for 10 min and then passed through a 0.2 µm syringe filter. A volume of 2 mL of the CFS was added to 2 mL of paraffin oil in a glass test tube and vigorously mixed for 2 min. The tubes were then incubated at room temperature for 24 h. The E_24_ value was calculated as shown in Eq. (3).

3 $${\text{E}}_{24}=\frac{{\text{H}}_{\text{EL}}}{{\text{H}}_{\text{S}}} \times 100$$

where E_**24**_ is the Emulsification Index (the percentage of crude oil biodegradation), H_EL_ is the height of the emulsified layer (mm), and H_S_ is the total height of the liquid column (mm).

### Residual crude oil analysis via GC–MS

The residual crude oil of the most potent oil-degrading isolates was analyzed to examine the resulting degradation compounds via the GC–MS technique at the National Research Center (NRC), Giza, Egypt. The residual crude oil extract obtained via n-hexane liquid–liquid extraction was concentrated to a volume of 0.2 mL. It was studied with a Hewlett Packard Agilent 5973 brand mass spectrometer coupled with the Hewlett Packard Agilent 6890 plus chromatograph. An aliquot (1 µL) of each sample was injected via splitless injection. The apparatus was equipped with an HP-5MS capillary column, with a 95% dimethylpolysiloxane phase, a length of 30 m, an internal diameter of 0.25 mm and a film thickness of 0.25 µm. Helium was used as the carrier gas. The temperature was increased at a rate of 10 °C/min (40 to 300 °C) with a hold time of 20 min and a total run time of 51 min. The data were collected on a Windows personal computer. The components’ identification was accomplished via computer search of user-generated reference libraries, incorporating mass spectra. Peaks were examined by single-ion chromatographic reconstruction to confirm their homogeneity. In some cases, when identical spectra have not been found, only the structural type of the corresponding component was proposed on the basis of its mass spectral fragmentation. Cochromatography of reference compounds was performed, when possible, to confirm GC retention times (Soumeya et al. 2022).

### Optimization of crude oil biodegradation

#### Plackett–Burman design (PB design)

To optimize the crude oil biodegradation by the most potent oil-degrading isolates, Plackett–Burman design (PBD) was used to screen significant factors that affect the deasphalted crude oil biodegradation (Plackett and Burman 1946). The independent variables were the initial concentration of crude oil (0.5–2%), actinobacterial inoculum (5–10 mm agar plug), incubation time (5–10 days), incubation temperature (25–40 °C), pH of the medium (6–9), and concentration of FeSO_4_ (0.005–0.015 g/l), yeast extract (0.05–0.15 g/l) and glucose (0.5–1 g/l) as demonstrated in Table 1. Each variable was examined at two levels: –1 for the low level and + 1 for the high level. The experimental range and variables used are shown in Supplementary Table S2. The values of the two levels were set according to our previous preliminary experimental results. A total of 12 experimental runs were performed via Design Expert trial® 12 (Stat-Ease, Inc., Minneapolis, MN, USA) to evaluate the analysis of variance (P < 0.05), the significance of each factor in the fitted equations, and the goodness of fit in each case. The levels were selected on the basis of the results of the experimental design, and the effect of each variable on crude oil biodegradation was determined via Eq. (4).

4 $$ Y = \beta_{0} + \sum \beta_{i} X_{i} $$

where Y is the measured response (the percentage of crude oil biodegradation), ${\beta }_{0}$ is the model intercept, ${\beta }_{i}$ is the linear factor coefficient, and ${X}_{i}$ is the level of the independent variable (El-Borai et al. 2016).

**Table 1:** Plackett–Burman design levels for the four independent variables showing the total of 12 sets of experimental work

Run	Crude oil (%)	Time (days)	Agar plug diameter (mm)				Temp. (°C)	pH	FeSO_4_ (g/L)	Yeast extract (g/L)	Glucose (g/L)
			A7	A12	B1	A2					
1	0.5	10	+ 1	−1	+ 1	+ 1	40	6	0.005	0.05	2
2	0.5	5	−1	+ 1	−1	+ 1	40	6	0.015	0.15	2
3	0.5	5	−1	−1	−1	−1	25	6	0.005	0.05	0.5
4	2	5	+ 1	+ 1	−1	+ 1	40	9	0.005	0.05	0.5
5	2	10	−1	−1	−1	+ 1	25	9	0.015	0.05	2
6	2	10	+ 1	−1	−1	−1	40	6	0.015	0.15	0.5
7	2	10	−1	+ 1	+ 1	+ 1	25	6	0.005	0.15	0.5
8	2	5	+ 1	+ 1	+ 1	−1	25	6	0.015	0.05	2
9	0.5	10	−1	+ 1	+ 1	−1	40	9	0.015	0.05	0.5
10	0.5	10	+ 1	+ 1	−1	−1	25	9	0.005	0.15	2
11	2	5	−1	−1	+ 1	−1	40	9	0.005	0.15	2
12	0.5	5	+ 1	−1	+ 1	+ 1	25	9	0.015	0.15	0.5

*Temp* temperature

#### Central composite designs (CCD)

According to the PB design results, the three most significant variables were selected to define the optimal conditions through the response surface methodology (RSM). Twenty runs of the CCD, with 1 axial point, 1 factorial point, and 6 center points (n_0_ = 6) were applied. Model significance, the prediction equation and the regression coefficient were statistically examined following ANOVA. Equation (5) represents the second-order polynomial equation used for fitting the results and analyzing the interaction between the selected factors.

5 $$\text{Y}={\upbeta }_{0}+{\upbeta }_{1}\text{A}+{\upbeta }_{2}\text{B}+{\upbeta }_{3}\text{C}+{\upbeta }_{12}\text{AB}+{\upbeta }_{13}\text{AC}+{\upbeta }_{23}\text{BC}+{\upbeta }_{11}{\text{A}}^{2}+{\upbeta }_{22}{\text{B}}^{2}+{\upbeta }_{33}{\text{C}}^{2}$$

where Y is the measured response (the percentage of crude oil biodegradation); ${\beta }_{0}$ is the model intercept; ${\beta }_{1}, {\beta }_{2}, and {\beta }_{3}$ represent the linear coefficients; ${\beta }_{12}, {\beta }_{13}, and {\beta }_{23}$ represent the interaction coefficients; ${\beta }_{11}, {\beta }_{22} and {\beta }_{33}$ represent the quadratic coefficients; and A, B and C represent the levels of the coded independent variables. Analysis of variance was performed, and response surface plots were generated via Design Expert trial® 12 (Stat-Ease, Inc., Minneapolis, MN, USA) (Popoola and Yusuff 2021; Singhania and Barmanray 2022).

#### Bioaugmentation pot assay

The most potent oil-degrading isolate was cultivated on ISP2 broth medium at 30 ± 2 °C in a Thermo Scientific MaxQ 4450 shaking incubator (120 rpm) for 1 week. The resulting cell suspension was used as an inoculum for the bioaugmentation pot assay. Nonpolluted garden soil was prepared according to Baoune et al. (2019). The pH of the soil used was 8.42. Two pots (25 cm diameter and 40 cm height) were filled with 4 kg of sieved silty clay loam soil. The soil samples were mixed well with 3% crude oil and left for 24 h. One pot was inoculated with the actinobacterium cell suspension to obtain a concentration of 2.4 × 10^6^ CFU/pot (i.e., 6 × 10^2^ CFU/g), which was mixed well to ensure uniform distribution of the cells. The other pot was inoculated with actinobacterium-free broth and regarded as the negative control. The pots were then incubated for 3 weeks at 55–65% humidity by the addition of sterile distilled water. Plastic saucers were placed beneath the pots to prevent water loss. The percentage of crude oil removal removal was determined as described previously, and the hydrocarbons in both soils were analyzed via GC–MS technique (Popoola et al. 2022).

#### Actinobacteria identification

All the isolated actinobacteria were initially identified phenotypically. The most potent oil-degrading isolate was subjected to both phenotypic and genotypic identification. Phenotypic identification was conducted via established keys from *Bergey's Manual of Systematic Bacteriology* and the International *Streptomyces* Project (I.S.P.) (Shirling and Gottlieb 1966; Madigan et al. 1997; Xu et al. 2007; Whitman 2012). The parameters examined included the following: (a) Culture morphology: assessed on different ISP media (Shirling and Gottlieb 1966), observing aerial and substrate mycelia, and overall growth appearance. (b) Spore chain morphology: Gram-stained cover-slip cultures. (c) Spore morphology and surface characteristics: Examined via a Thermo Fisher Scientific Quanta FEG 250 scanning electron microscope (SEM) and a JEM-2100 HRT transmission electron microscope (TEM) at NRC, Egypt. (d) Biochemical properties: These properties included the production of diffusible pigments, the ability to grow on Czapek’s agar, tolerance to various concentrations of sodium chloride (2.5, 5, 7.5 and 10%), and sensitivity to 50 µg mL^−1^ streptomycin via agar disc diffusion. (e) Antimicrobial activity: Determined via the Spectra-Plak method (Barka et al. 2016; Muthukrishnan et al. 2020) by streaking the actinobacterial culture onto agar plates and incubation at 30 ± 2 °C for 5 days, after which the plates were subsequently seeded with a perpendicular streak of target microorganisms and incubated for another 24 h (in the case of bacteria and yeasts) or 5 days (in the case of molds). After incubation period, zones of inhibition (ZOIs) were observed.

The genotypic identification of the most effective oil-degrading actinobacterium was performed on the basis of the amplification and sequencing of its 16S rRNA gene. DNA isolation and amplification were conducted at the Animal Health Research Institute (AHRI), Giza, Egypt. DNA was isolated via a QIAamp DNA Mini Kit (Qiagen Inc.). Polymerase chain reaction (PCR) was performed via a T3 thermal cycler (Biometra) and oligonucleotide primers designed for the order Actinomycetales: 243F (5'-GGATGAGCCCGCGGCCTA-3') and A3R (5'-CCAGCCCCACCTTCGAC-3') (Metabion, Germany) (Monciardini et al. 2002). The amplified DNA product was separated using agarose gel electrophoresis (using ABgarose, Tris borate EDTA (TBE) electrophoresis buffer, and an Alpha Innotech gel documentation system) and visualized with ethidium bromide (Sigma) (Sambrook et al. 1989). The purified PCR product was sequenced in both the forward and reverse directions using Applied Biosystems® 3130/3130xl Genetic Analyzers at ELIM Biopharmaceuticals, USA. The BigDye™ Terminator v3.1 Cycle Sequencing Kit (Applied Biosystems™, California) was utilized. The obtained partial sequence was compared against sequences in the National Center for Biotechnology Information (NCBI) database (http://www.ncbi.nlm.nih.gov) via the Basic Local Alignment Search Tool (Nucleotide) (BLASTN) algorithm. The sequence was subsequently submitted to NCBI GenBank and assigned an accession number. A phylogenetic tree between the tested actinobacterium and its closest related strains found in GenBank was constructed via the neighbor-joining (NJ) method using MEGA11 (Tamura and Nei 1993; Thompson et al. 1994).

### Statistical analysis

The results of the gravimetric analysis and emulsification index were analyzed using Origin software, version 6.1 (OriginLab), and the means and standard deviations are illustrated in the figures.

## Results and discussion

### Soil analysis

The physicochemical analysis of the three collected soil samples revealed that all the soil samples were alkaline and that their pH ranged from 8.27 to 8.87. S-MOTOR soil presented the highest EC and organic matter content (15.36), followed by S-PEST soil, as both were collected from rural areas, whereas S-CRUDE soil presented the lowest organic matter content (6.72%) as it was collected from a petroleum extraction site in the desert. The soil textures of S-CRUDE, S-MOTOR and S-PEST soils were silt loam, sandy loam, and loamy sand, respectively. The highest fluorine and zinc contents were estimated in S-CRUDE soil, while S-MOTOR soil presented the highest chlorine, sulfate and potassium contents, and S-PEST soil presented the highest copper content. Nitrite, phosphate, cadmium, lead, and chromium were not detected in any of the soil samples, whereas nitrate was detected only in S-MOTOR soil. The results of the physicochemical analysis of the polluted soil samples are shown in Supplementary Table S3.

The analysis of the GC–MS spectra revealed that some hydrocarbons, such as eicosane, heptadecane, hexacosane, hexatriacontane, nonadecane, octacosane, pentadecane, tetracosane, tetracosane, tetratriacontane, and tricosane, were present in all soil samples. The GC–MS analysis of S-CRUDE soil revealed 29 compounds, and the total peak area was 100%. The major compounds were docosane (15.4854%), which is a component of crude oil, and hexacosane (9.4383%), nonadecane (9.1035%), tricosane (8.1255%), octadecane (7.5908%) and nonacosane (6.7099%), which were detected after crude oil treatment in the subsequent biodegradation experiments. They represented 56.4534% of the total peak area. The GC–MS analysis of S-MOTOR soil showed 26 compounds and the total peak area was 100%. The major compounds were hexatriacontane (13.7273%), tricosane (12.8559%), hexacosane (12.1078%), z-14-nonacosane (11.7765%) and octacosane (11.6041%), and they represented 62.0716% of the total peak area. Finally, the GC–MS analysis of S-PEST soil revealed 35 compounds, and the total peak area was 100%. The major compounds were isochiapin B (30.49%), 2-methyl-1-hexadecanol (16.17%), dodecyl acrylate (14.42%) and 1,2-benzene dicarboxylic acid (9.35%). They represented 70.43% of the total peak area. The GC–MS chromatograms of the polluted soil samples are shown in Supplementary Figure S1, while the compounds detected in the GC spectra of the polluted soil samples are listed in Supplementary Table S4.

### Biodegradation of crude oil

Fifteen actinobacterial isolates were isolated from S-CRUDE soil (assigned letter A), six from S-MOTOR soil (assigned letter B), and seven from S-PEST soil (assigned letter C) on MSM agar with 2% crude oil. All 28 purified actinobacteria revealed variable oil biodegradable potential. The residual crude oil was examined quantitatively via gravimetric analysis and qualitatively via GC–MS analysis.

### Gravimetric analysis of residual crude oil

**Fig. 1:**
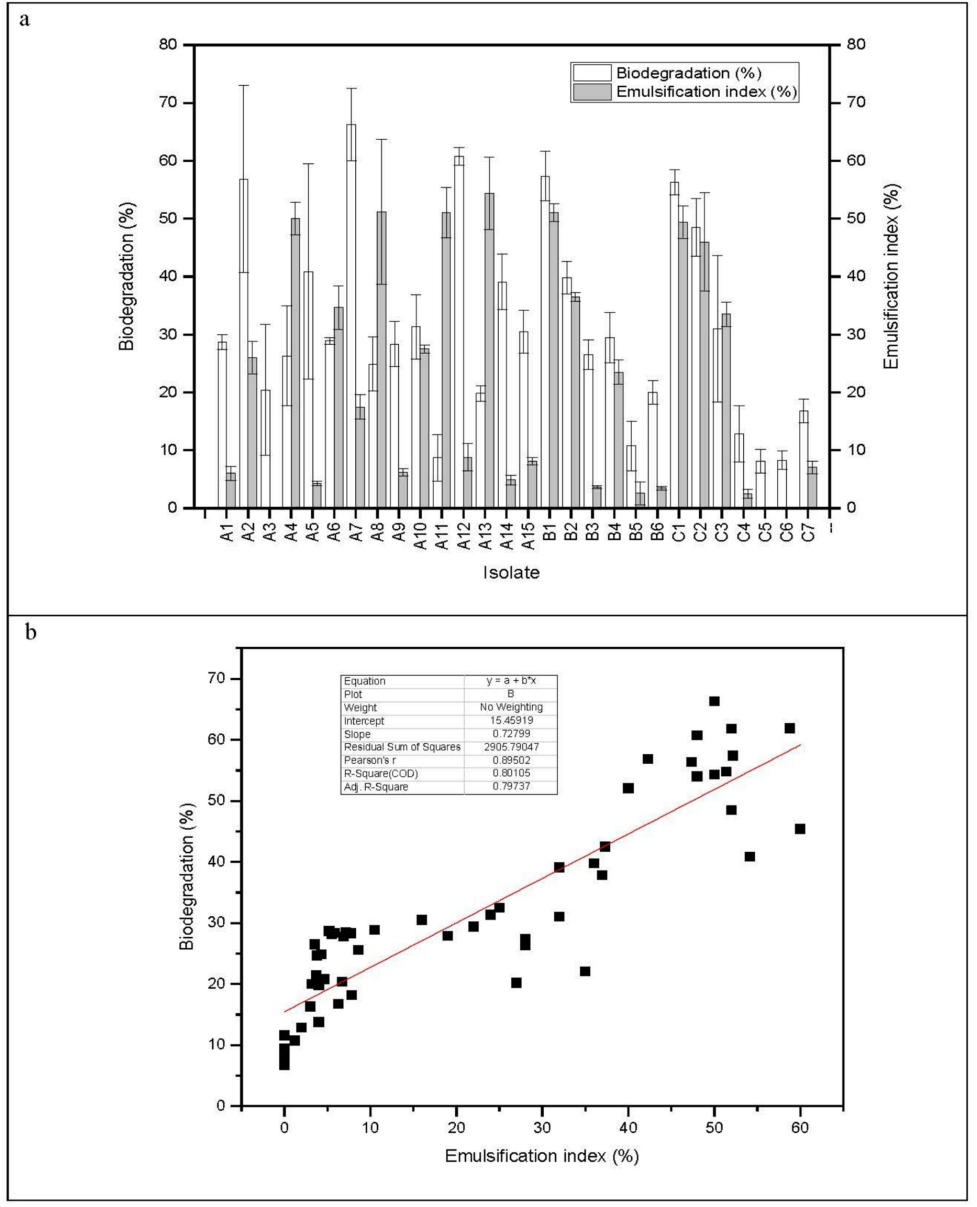
Percentage of biodegradation of crude oil and emulsification index for each isolate. **a** Normal plot showing variation in the biodegradation capabilities and emulsification indices of the tested actinobacteria and **b** linear fit showing a positive correlation between the emulsification index and the percentage of biodegradation of crude oil

The biodegradation percentage was found to vary from 8.12 ± 2.00 to 66.28 ± 6.25%. The most bioactive isolate was isolate A7 followed by isolate A12 (60.75 ± 1.48%), and both were indigenous to the S-CRUDE soil contaminated with crude oil. On the other hand, the isolate that showed the lowest biodegradation efficiency was isolate C5, which was exogenous to S-CRUDE soil (Fig. 1a).

To provide a comprehensive overview of the application of actinobacteria in crude oil degradation, compared with that of the other bacteria and fungi, findings from some pertinent references revealed variable results. A study involving three *Streptomyces* species isolated from soil in Algeria reported that petroleum and naphthalene could be removed up to 85.23% after 21 days of incubation (Ferradji et al. 2014). Another study by Olajuyigbe and Ehiosun (2016) reported that *Actinomyces viscosus* and *Actinomyces israelii* could degrade crude oil with efficiencies of 87% and 80%, respectively. Additionally, Ehiosun and Usman (2018) reported that *Streptomyces albus* broke down more than 50% of crude oil during the log phase of growth. This percentage increased to above 80% after 10 days for all concentrations of crude oil they tested in their study (1–7.5%). Moreover, Abbas et al. (2022) examined 18 bacterial isolates that belong to the genera *Pseudomonas*, *Alcaligenes*, *Bacillus*, *Dietza* and *Streptomyces*, and 4 fungal isolates belonging to the genus *Aspergillus*. Among the tested bacteria, *Bacillus subtilis* RGB6 showed the highest biodegradation percentage (68.3%), whereas *Aspergillus terreus* RGF3 and *Aspergillus terreus* RGF1 showed the maximum percentages among the tested fungi (86.5 and 70.3%, respectively). Furthermore, the genera *Bacillus*, *Burkholderia*, *Providencia* and *Klebsiella* were examined in a study of diesel biodegradation and achieved 40–52% degradation (Ali et al. 2021). Studies that included mixed cultures and microbial consortia activity in crude oil degradation have also been reported. Varjani et al. (2020) reported that bioaugmented microcosms containing hydrocarbon-degrading bacteria remediated soil polluted with oily sludge and removed up to 82.13% of oil on the 56th day. Also, Muthukumar et al. (2022) examined the crude oil biodegradation capability of two strains of *Pseudomonas aeruginosa* and a mixed consortium. They reported that the biodegradation of 1% crude oil in MSM reached 50% and 86% by strains PP3 and PP4, respectively, after incubation for 15 days. Moreover, Bajelani et al. (2023) tested a mixed culture of two strains of bacteria and one yeast strain, *Arthrobacter citreus*, *Bacillus thuringiensis* and *Candida catnulata*. The results confirmed that this mixture could make a biofilm and degrade 75% of the diesel in 10 days.

### Emulsification index (E24)

Biosurfactants are “active compounds produced at the microbial cell surface or excreted, and reduce surface and interfacial tension” (Costa et al. 2018). The emulsification index assay is an indirect technique for screening microbial biosurfactant production. Biosurfactant production is a property of hydrocarbon-degrading microorganisms (Ilori et al. 2005). These molecules facilitate oil uptake by microorganisms and emulsify hydrocarbons, increasing their biodegradability (Karlapudi et al. 2018). The emulsification indices of the tested actinobacteria are shown in Fig. 1b. The biosurfactant production ranged from 0 to 54.40 ± 6.22%, and the isolate with the highest biosurfactant production was isolate A13, followed by A8, A11 and B1. The most potent isolate for crude oil biodegradation had E_24_ value of 17.5 ± 2.12%, whereas isolate A11, which had E_24_ value of 51.09 ± 4.36%, exhibited low crude oil biodegradation (8.75 ± 4.03%). On the other hand, the lowest isolates for regarding crude oil degradation (C5, C6 and A11) had zero E_24_ values, which indicates that no biosurfactant was produced. The most likely explanation for this result is that many authors have reported that most, but not all crude oil degraders produce extracellular biosurfactants (Mohanty et al. 2013; Karlapudi et al. 2018).

The scatter plot and Pearson correlation (r = 0.89502) suggest a strong positive correlation between the emulsification index and the biodegradation percentage of crude oil as the value is close to one indicating a strong degree of linear dependence (Table 2).

**Table 2:** Pearson correlation value calculated from the linear fit plot

Value	N	Mean	SD	Sum	Min	Max
Biodegradation efficiency (%)	56	21.78	20.04	1219.92	0.00	60.00
Emulsification index (%)	56	31.32	16.30	1753.81	6.70	66.28
Pearson Correlation Coefficient (r) (%)	0.89502					

Mona Othman (2020) isolated 15 bacterial strains from oil-contaminated areas, 5 of which could use petroleum as a sole carbon source. They reported that *Streptomyces* sp. MB9 was the most effective for crude oil breakdown, displaying the highest emulsifying capacity, which indicated the highest biosurfactant production. Additionally, Srimathi et al. (2024) examined 39 crude oil-degrading bacteria that were isolated from different soils in India. The E_**24**_ of these strains ranged from 22.35 to 61.20% and the highest isolate was *Bacillus licheniformis* EBPL0613-F2. Furthermore, Kieu et al. (2024) reported that the emulsification index (E_24_) of *Bacillus megaterium* VTVK15 was 51.8%, and the strain showed a total petroleum hydrocarbon degradation (TPH) efficiency of 72% after 14 days.

Nagime et al. (2023a) demonstrated a sustainable biorefinery approach using *Enterococcus gallinarum* to produce biosuccinic acid from palm oil mill effluent, achieving 23.7 g/L with optimized nutrients and 19.1 g/L by utilizing biogas-derived CO_2_. Additionally, Nagime et al. (2023b) demonstrated enhanced succinic acid production (up to 50 g/L with 0.79 g/g yield) from lignocellulosic hydrolysate via the use of metabolically engineered *Actinobacillus succinogenes* and optimized fed-batch fermentation, providing a sustainable pathway for bio-based chemical synthesis. Succinic acid is not usually a direct reactant added to break down crude oil. Instead, its derivatives (alkylsuccinates and arylalkylsuccinates) are crucial initial intermediates formed during the anaerobic activation of both aliphatic and aromatic hydrocarbons via the fumarate addition pathway. Consequently, these succinate derivatives also serve as biomarkers indicating active anaerobic crude oil biodegradation in contaminated environments. Furthermore, succinic acid itself is a fundamental intermediate in microbial metabolism, meaning that it is involved in the broader metabolic processing of hydrocarbon breakdown products (Jarling et al. 2015; Rabus et al. 2016; Baensch et al. 2025).

### GC–MS analysis of untreated and residual crude oil

The GC–MS spectrum of the untreated crude oil sample (negative control) revealed 35 compounds (Supplementary Figure S2a). The total peak area of the separated compounds was 100%, and the major compounds found were docosane (23.60%), docosyltrichlorosilane (17.76%), 2,2,4,9,11,11-hexamethyl, dodecane (10.86%), and 1-dodecane (10.15%), which represented 62.37% of the total peak area. The overall composition indicated a greater presence of long-chain hydrocarbons, which are typical in crude oil.

GC–MS analysis of the residual crude oil after treatment with the bioactive isolates A2, A7, A12, and B1, which presented the highest biodegradation percentages according to gravimetric analysis (56.85 ± 16.19, 66.28 ± 6.25, 60.75 ± 1.48 and 57.38 ± 4.28%, respectively), was also performed (Supplementary Figure S2b, c, d and e). The compounds detected in the analysis of the GC spectra of untreated crude oil only, untreated crude oil and all treatments, untreated crude oil and some treatments, and treated crude oil only are shown in Supplementary Tables S5, S6, S7, and S8 respectively. The structures of the major compounds found in untreated crude oil and crude oil treated with isolates A2, A7, A12, and B1 are shown in Supplementary Figure S3.

The crude oil treated with isolate A2 was composed of 36 compounds (Supplementary Figure S2b). The total peak area of the detected compounds was 100%. The major compounds detected were hexadecane (19.87%), which was also found in untreated crude oil, and 3,7,11-trimethyl-1-dodecanol (16.05%), which was not found in untreated crude oil, suggesting that A2 produced the latter as a result of the oil degradation process. The total major compounds represented 35.92% of the total peak areas. There was a marked decrease in docosane (3.24%) and 1-tetradecanol (2.32%), suggesting that A2 may promote the breakdown of long-chain hydrocarbons.

The crude oil treated with isolate A7 contained 35 compounds (Supplementary Figure S2c). The total peak area of the constituents was 100%. The major compound that was also found in untreated crude oil was docosane (10.35%), whereas those that were not detected in untreated crude oil were 7-methylpentadecane (20.22%), pentacosane (12.73%) and nonadecane (10.04%). The major compounds represented 53.34% of the total peak areas. Treatment with A7 further reduced the content of docosane and 1-tetradecanol (0.53%). Interestingly, 2,6,10-trimethyltetradecane was present at a lower percentage (0.93%) than A2 (6.78%), indicating a different metabolic pathway or efficiency.

The crude oil treated with isolate A12 consisted of 31 substances (Supplementary Figure S2d). The total peak area of the obtained compounds was 100%. The major compounds that were also found in untreated crude oil were 2,6,10-trimethyltetradecane (21.06%) and docosane (9.67%), On the other hand, those that were not detected in untreated crude oil were pentacosane (17.60%) and pentadecane (8.53%). This result confirmed the ability of isolate A12, as well as isolates A7 and A2, to transform the oil structure. The total major compounds represented 56.86% of the total peak areas. Treatment with A12 significantly increased the content of 2,6,10-trimethyltetradecane (21.06%), while that of docosane drops to 9.67%. This suggests that A12 is particularly effective in altering the hydrocarbon profile, potentially leading to more favorable compounds for further processing.

The crude oil treated with isolate B1 contained 33 compounds (Supplementary Figure S2e). The total peak area of the detected compounds was 100%. The major compounds that were not found in untreated crude oil were heptadecane (13.07%) and octadecane (12.02%), whereas those found in untreated crude oil were docosane (10.31%) and 9-octadecenoic acid (Z) (14.89%). Actinobacterium B1, as well as A2, A7, and A12, can utilize crude oil as a sole source of carbon and produce new compounds. The total major compounds represented 50.29% of the total peak areas. Compared to control (untreated crude oil), treatment with B1 resulted in a moderate presence of docosane (10.31%), a notable decrease in 2,6,10-Trimethyltetradecane, and increase in 9-octadecenoic acid (Z), This finding indicated that B1 also contributed to the transformation of crude oil but not as effectively as the other isolates did.

The major compounds in the untreated and treated crude oils were all open-chain compounds. The compounds that appeared in the treated residual crude oil but not in the untreated crude oil were produced via the tested actinobacteria. Similarly, the disappearance or decrease in the peak areas of some compounds in treated oil—compared with those in the control—proved the utilization and/or degradation of these components by the tested isolate(s). Some compounds detected in crude oils treated with four potential actinobacteria, A2, A7, A12, and B, have important applications in different industries, including the fragrance, cosmetic, pesticide, plastic, rubber, food, and pharmaceutical industries (Table 3). Each of the four treatments significantly altered the composition of the crude oil, reducing the abundance of long-chain hydrocarbons such as docosane while sometimes increasing the presence of branched hydrocarbons such as 2,6,10-trimethyltetradecane. Each microorganism exhibited different efficiencies and pathways in transforming crude oil. These changes are crucial for understanding how microbial treatments can increase the processing and utilization of crude oil, potentially leading to more efficient extraction and refining processes.

**Table 3:** Uses of some of the compounds detected in the GC–MS analysis of residual crude oil

No	Compound	Isolate(s) producing these compounds	Application(s)	Reference
1	9,12-Octadecadienoic acid (Z,Z)-, methyl ester Another name: Linoleic acid, methyl ester	A2	Emollient and fragrance components	NCBI (2024c)
2	1,1'-Oxybis, decane	A7	Cosmetic ingredients	NCBI (2024d)
3	1,4-Butanediamine	A2	Pesticides	NCBI (2024e)
4	2-(Octadecyloxy)ethanol	B1	Plastics, rubber, and cosmetics (Cleansing; Gel forming; Surfactant; Refatting; Emulsifying)	NCBI (2024f)
5	2,3-Dihydroxypropylester, hexadecanoic acid	B1	Biochemical research and cosmetics (emollient)	NCBI (2024g)
6	2-Methyl, pentadecane	B1	Cosmetic ingredients (skin conditioning, emollient, and solvent)	NCBI (2024h)
7	3,4-Dimethyl, heptane	A12	Solvent	NCBI (2024i)
8	3,4-Dimethyl, octane	A7	Solvent	NCBI (2024j)
9	4,5-Dimethyl, nonane	A7	Solvent	NCBI (2024b)
10	Aspartame	A12	Food additives (sweetener), and cosmetics (masking)	NCBI (2024k)
11	Dibutyl ester, nonanedioic acid	B1	Plastics and rubber	NCBI (2024l)
12	Eicosane	A12 & B1	Fragrance ingredient	NCBI (2024m)
13	Heptadecane	A7 & B1	Fragrance ingredient	NCBI (2024n)
14	Nonadecane	A7	Fragrance ingredient	NCBI (2024o)
15	Octadecane	A7 & B1	Fragrance ingredients and cosmetics (skin conditioning, emollient, and solvent)	NCBI (2024p)
16	Octadecanoic acid (other name: stearic acid)	B1	Cosmetic ingredients (cleansing, emulsion stabilizing, refatting, and emulsifying), food additives (emulsifier or emulsifier salt, flavouring agent or adjuvant, formulation aid, lubricant or release agent, masticatory substance, surface-active agent), fragrance ingredient, and plastics	NCBI (2024q)
17	Paromomycin	A12	Pharmaceuticals (broad-spectrum antibiotic)	NCBI (2024r)
18	Pentadecane	A12 & B1	Fragrance ingredient	NCBI (2024s)
19	Tetradecane	A2 & B1	Fragrance ingredient	NCBI (2024t)
20	Tricosane	A7	Fragrance ingredient	NCBI (2024a)
21	Tridecanol	A7 & A12	cosmetic ingredient (emulsion stabilizing, viscosity controlling, emollient, refatting), and fragrance ingredient	NCBI (2024u)

### Optimization of crude oil biodegradation

#### Plackett–Burman design (PB design)

Plackett–Burman’s (PB) design is a powerful statistical screening designed to select significant factors among many key factors, reduce errors and cost-effectively obtain results. The experimental design and response for each trial are described in Table 4. The crude oil removal percentage was found to vary from 32.22 to 60.40% among the 12 runs. The maximum degradation was observed at run number 9, followed by runs 2, 6, and then 4, whose outcome removal percentages were 60.40, 56.60, 55.65, and 53.95%, respectively. The Model F-value of 1038.54 implies that the model is significant. P-values less than 0.0500 indicate that the model terms are significant. In our case, A, B, C, E, F, G, H, J, K, and L are significant model terms (Table 5).

**Table 4:** Plackett–Burman experimental design and response for each run

Std	Run	Crude oil removal%	
		Actual	Predicted
2	1	43.60	43.51
6	2	56.60	56.69
12	3	49.20	49.11
3	4	53.95	53.86
8	5	43.00	42.91
9	6	55.65	55.74
1	7	41.35	41.44
11	8	33.00	33.09
4	9	60.40	60.49
10	10	32.20	32.29
7	11	39.10	39.01
5	12	51.40	51.31

**Table 5:** ANOVA for the selected factorial Plackett–Burman model

Source	Sum of squares	df	Mean square	F-value	p-value	
Model	954.16	10.00	95.42	1038.54	0.02	Significant*
A-Hydrocarbon	189.21	1.00	189.21	2059.43	0.01	
B-Incubation time	111.33	1.00	111.33	1211.70	0.02	
C-Isolate A7	64.17	1.00	64.17	698.47	0.02	
E-Isolate B1	22.55	1.00	22.55	245.44	0.04	
F-Isolate A2	20.15	1.00	20.15	219.32	0.04	
G-Temperature	56.99	1.00	56.99	620.25	0.03	
H-pH	108.90	1.00	108.90	1185.33	0.02	
J-Ferrous sulphate	115.63	1.00	115.63	1258.56	0.02	
K-Yeast extract	177.49	1.00	177.49	1931.81	0.01	
L-Glucose	87.75	1.00	87.75	955.10	0.02	
Residual (D-Isolate A12)	0.09	1.00	0.09			
Cor Total	954.25	11.00				

*Significant at 5% level (P < 0.05), *dF* degree of freedom, *P* corresponding level of significance

**Fig. 2:**
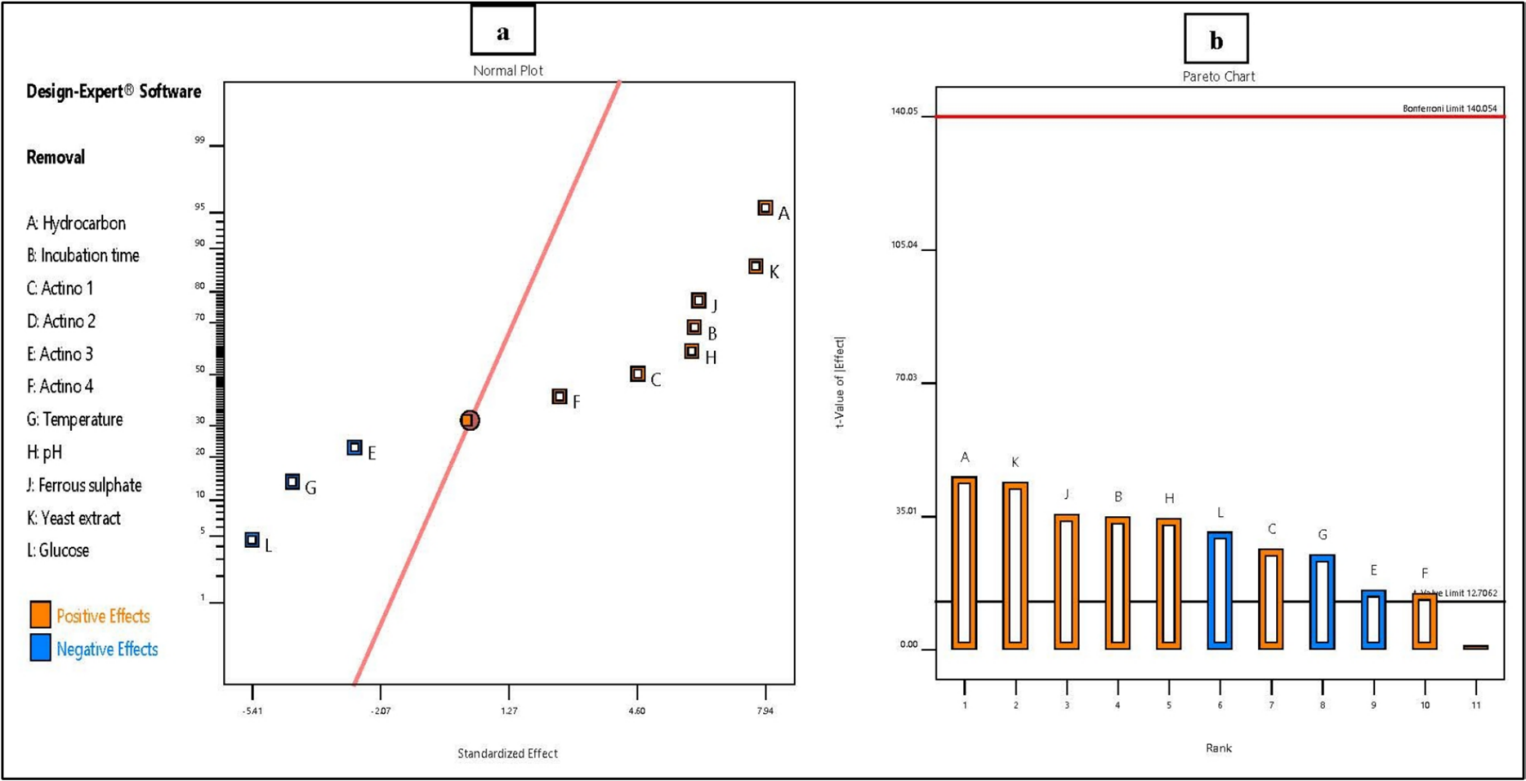
Positive and negative factors influencing the biodegradation of crude oil. **a** Normal probability plots, **b** Pareto chart showing positive (colored orange) and negative effects (colored blue) of the different tested factors on crude oil biodegradation. Actino 1, Actino 2, Actino 3, and Actino 4 represent the agar plug diameter (mm) of isolates A7, A12, B1, and A2, respectively

The results in the normal plot of the standardized effects from Plackett–Burman showed that A- concentration of crude oil, B- incubation time, C- actinobacterial isolate A7, F- actinobacterial isolate A2, H- pH, J- ferrous sulphate and K- yeast extract had positive impacts on the removal of crude-oil, whereas E- actinobacterial isolate B1, G- temperature, and L- glucose adversely affected the percentage of removal (Fig. 2). The correlation equation that describes the relationship between the analyzed variables and the tested response (percentage of removal of crude oil) using the coefficients obtained from Plackett–Burman design is shown in Eq. (6).

6 $$\text{R}=15.85+5.29\text{A}+1.22\text{B}+ 2.31\text{C}-1.37\text{E}+1.30\text{F}-0.29\text{G}+2.01\text{H}+620.83\text{J}+76.92\text{K}-3.61\text{L}$$

where R represents the removal of crude oil (%), A represents the concentration of crude oil (%), B represents the incubation time (days), C represents plug diameter of isolate A7 (mm), E represents the agar plug diameter of isolate B1 (mm), F represents the agar plug diameter of isolate A2 (mm), G represents the temperature (°C), H represents the pH, J represents the concentration of ferrous sulfate (%), K represents the concentration of yeast extract (g/l), and L represents the concentration of glucose (%). The equation expressed in terms of actual factors could serve as a tool for estimating predictions regarding the response at specified levels of each factor.

The coefficient estimate represents the expected change in response per unit change in factor value when all remaining factors are held constant. The intercept in an orthogonal design is the overall average response of all the runs. The coefficients are adjusted around that average on the basis of the factor settings. When the factors are orthogonal, the VIFs are 1; VIFs greater than 1 indicate multicollinearity. The higher the VIF is, the more severe the correlation of factors. As a rough rule, VIFs less than 10 are tolerable. The coefficients in terms of coded factors are shown in Supplementary Table S9.

The results of the ANOVA test and calculated t-test revealed that isolate A7 was the most effective in crude oil degradation, and the initial concentration of crude oil and the concentration of yeast extract were the most significant variables affecting crude oil biodegradation (Table 5). Therefore, they were subjected to response surface methodology (RSM) to confirm the optimized conditions for a high percentage of crude oil biodegradation.

### Central composite design (CCD)

A fundamental component of the RSM is the Central Composite Designs (CCD) model. This optimization model is more accurate and does not require a three-level factorial experiment to create a second-order quadratic model (Granato and de Araújo Calado 2014). The three most significant variables identified via the PB design, A, crude oil concentration (%), B, yeast extract concentration (g/l), and C, inoculum size of isolate A7 (mm agar plug), were selected for optimization of crude oil biodegradation via the RSM through CCD.

The predicted and actual responses for each run are also illustrated in Table 6. The maximum degradation was observed in run number 7, followed by runs 15, 6, 13 and 9, where the crude oil removal percentages were 78.50, 72.50, 70.00, 69.50, and 69.33%, respectively. The optimum point for crude oil degradation predicted by the central composite design of the Design-Expert software was achieved in run 3, where the concentration of crude oil, the concentration of yeast extract, and inoculum size of isolate A7 were 3%, 0.15 g/L and 25 mm, respectively. The predicted values were contrasted with the observed experimental data to validate the previously discussed points. The results revealed 70% removal of crude oil compared with the model predicted percentage (74.43%), which indicated a relatively small error between the predicted and experimental values. This affirms the ability of the developed models to accurately predict the impact of strain A7 isolated from petroleum hydrocarbon-polluted soil as an ideally suitable actinobacterium for adequate crude oil degradation. As a result, strain A7 is a potential fighter against oil contamination of soil and other ecosystems.

**Table 6:** Central composite designs (CCD) levels for the 3 variables showing a total of 20 runs and the responses for each run

std	Run	Crude oil (%)	Yeast extract (g/L)	Agar plug diameter of isolate A7 (mm)	Response (% Crude oil removal)	
					Actual	Predicted
14	1	2.00	0.10	30.11	63.20	58.89
13	2	2.00	0.10	4.89	50.50	52.65
11	3	2.00	0.02	17.50	50.60	52.81
19	4	2.00	0.10	17.50	52.00	55.77
12	5	2.00	0.18	17.50	60.50	58.73
4	6	3.00	0.15	10.00	70.00	69.68
10	7	3.68	0.10	17.50	78.50	79.32
1	8	1.00	0.05	10.00	42.30	38.15
2	9	3.00	0.05	10.00	69.33	66.16
17	10	2.00	0.10	17.50	53.10	55.77
9	11	0.32	0.10	17.50	28.80	32.21
5	12	1.00	0.05	25.00	42.66	41.86
6	13	3.00	0.05	25.00	69.50	69.87
16	14	2.00	0.10	17.50	53.60	55.77
8	15	3.00	0.15	25.00	72.50	73.39
15	16	2.00	0.10	17.50	54.30	55.77
7	17	1.00	0.15	25.00	44.80	45.37
20	18	2.00	0.10	17.50	57.20	55.77
3	19	1.00	0.15	10.00	43.87	41.67
18	20	2.00	0.10	17.50	58.10	55.77

The results were analyzed via ANOVA to determine the individual effect of each factor and the interaction between the selected variables (Table 7). The Model F-value was 33.95 which implies that the model was significant. Factors A (initial crude oil concentration) and C (inoculum size of isolate A7) are significant model terms as their P-values were less than 0.0500. The F-value was low (2.15) and the P-value was high (0.21), which implies that the lack of fit is not significant relative to the pure error in the present study. A non-significant lack of fit is preferred.

**Table 7:** ANOVA for the selected quadratic CCD model

Source	Sum of Squares	df	Mean Square	F-value	p-value	
Model	2790.49	9.00	310.05	33.95	< 0.0001	Significant*
A-Hydrocarbon	2679.24	1.00	2679.24	293.33	< 0.0001	
B-Yeast extract	42.28	1.00	42.28	4.63	0.06	
C- Isolate A7	46.94	1.00	46.94	5.14	0.05	
AB	0.00	1.00	0.00	0.00	1.00	
AC	0.24	1.00	0.24	0.03	0.88	
BC	1.05	1.00	1.05	0.12	0.74	
A^2^	0.06	1.00	0.06	0.01	0.94	
B^2^	5.31	1.00	5.31	0.58	0.46	
C^2^	16.40	1.00	16.40	1.80	0.21	
Residual	91.34	10.00	9.13			
Lack of fit	62.31	5.00	12.46	2.15	0.21	Not significant
Pure error	29.03	5.00	5.81			

*Significant at 5% level (P < 0.05), *dF* degree of freedom, *P* corresponding level of significance

The correlation equation that describes the relationship between the analyzed variables and the tested response (percentage of removal of crude oil) using the coefficients obtained from the CCD is shown in Eq. (7).

7 $$\text{R}=29.27+13.87\text{A}-30.11\text{B}- 5.59\text{C}-0.10\text{AB}+0.23\text{AC}+9.67\text{BC}-0.0654{\text{A}}^{2}+242.91{\text{B}}^{2}+1.901{\text{C}}^{2}$$

where R is the percentage of crude oil removal (%), A is the concentration of crude oil (%), B is the concentration of yeast extract (g/L), and C is the inoculum size of isolate A7 (plug diameter, mm). The equation expressed in terms of actual factors could function as a tool for estimating predictions while considering the response at specified levels of each factor. The model R^2^ was 0.9683. The adjusted R^2^ (0.9398) was in reasonable agreement with the predicted R^2^ (0.8212), i.e., the difference was less than 0.2. The Adeq precision, the signal-to-noise ratio, was 22.045, i.e., greater than 4, which is desirable and indicates an adequate signal.

**Fig. 3:**
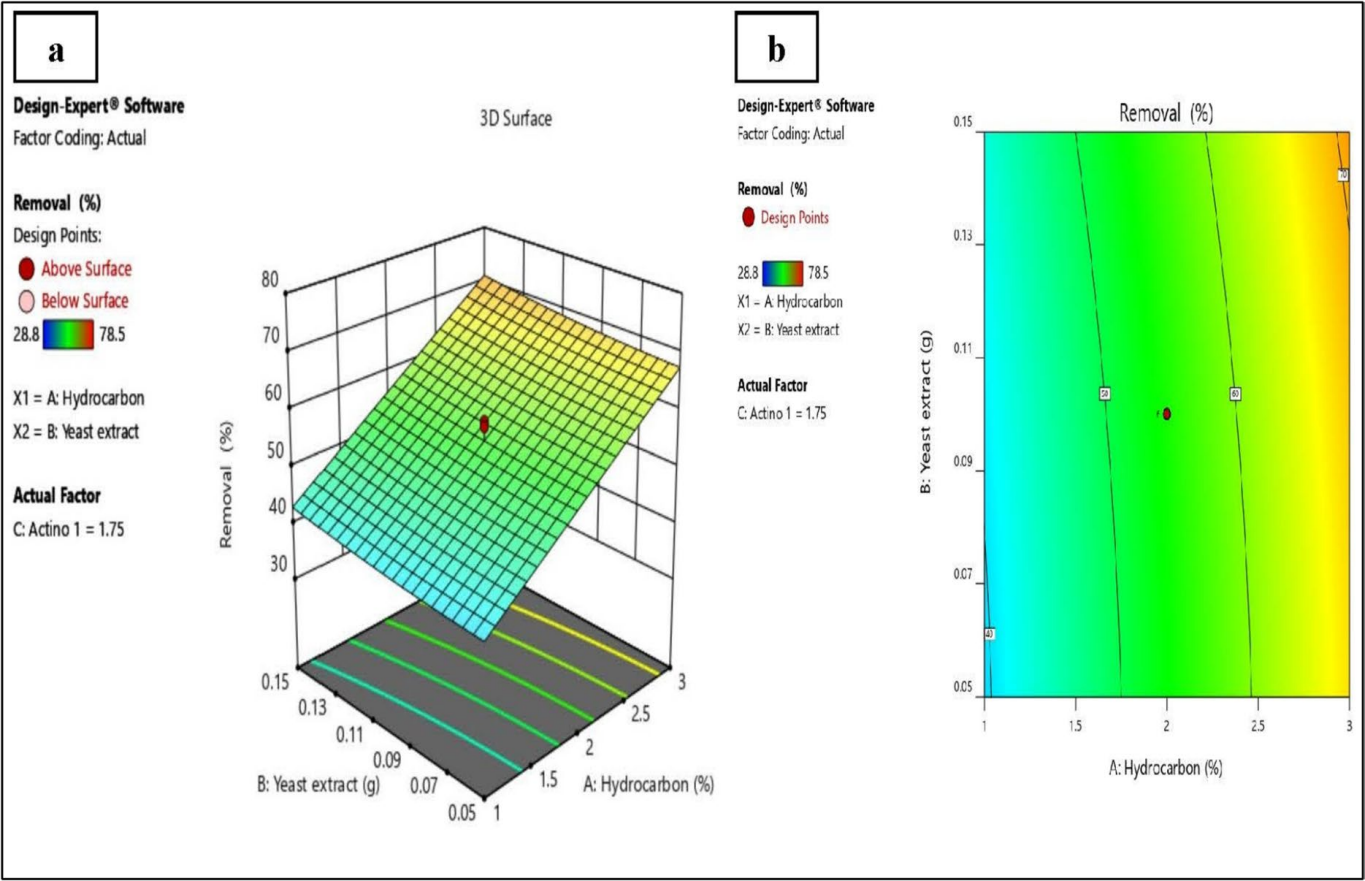
CCD model graphs developed by Design Expert ® software. **a** 3D surface plot, **b** contour plot for the change in crude oil degradation under different concentrations of hydrocarbon and yeast extract with fixed inoculum (1.75 mm agar plug of A7 culture), showing the effect of these variables on crude oil degradation using isolate A7

To visualize the relationship between the experimental variables and responses, 3D plots are generated from the models (Korayem et al. 2015). The obtained CCD model graphs are shown in Fig. 3. Based on this figure, with increasing the concentrations of hydrocarbon and yeast extract, crude oil degradation was observed to be smoothly increasing.

### Bioremediation of crude oil through bioaugmentation (pot assay)

Gravimetric analysis of residual crude oil in the soil samples in the bioaugmentation pot assay was performed after 21 days. The percentage of crude oil removal was 33.3 and 92% in case of the control soil and the soil sample treated with a cell suspension of isolate A7, respectively. A substantial improvement in crude oil degradation was observed with the introduction of the bacterial cell suspension, resulting in a 58.7% increase in crude oil removal efficiency. This finding provides a strong basis for its application in accelerating environmental cleanup.

The control soil and the soil inoculated with isolate A7 were analyzed via GC–MS after 1, 2 and 3 weeks of incubation. The results of the GC–MS analysis are shown in Supplementary Figure S4 and the compounds detected in the GC–MS spectra of the control and treated soils are listed in Supplementary Table S10. The GC–MS analysis of the control soil revealed 37, 46 and 39 compounds after 1, 2 and 3 weeks, respectively. The major compounds were different except for heptadecane after 1, 2 and 3 weeks. The major components after 1 week were hexatriacontane (16.84%), triacontane (9.96%), heptadecane (7.58%), and 4-methyl, tridecane (6.92%), which represented (41.3%) of the total peak area. The major constituents after 2 weeks were nonadecane (7.07%), pentadecane (6.38%), hexadecane (6.15%), and tetradecane (5.91%), accounting for 25.51% of the total peak area. After 3 weeks, the major compounds were tetracosane (11.06%), docosane (10.65%), heptadecane (8.55%), and octadecane (7.30%), which represented (37.56%) of the total peak area.

The GC–MS analysis of soil treated with isolate A7 revealed 30 compounds after 1 week and 36 during the 2nd and 3rd weeks. After 1, 2 and 3 weeks, the major compounds were different except for docosane and tetratetracontane, which were the original components of the crude oil used. The major compounds found after the 1st week were docosane (18.49%), tricosane (14.36%), tetratetracontane (8.59%), and hexacosane (7.37%), which represented (48.81%) of the total peak area. The major compounds detected after the 2nd week were docosane (27.44%), octadecane (6.74%), tetratriacontane (6.45%), and pentacosane (5.53%), accounting for 46.16% of the total peak area. Finally, the major components obtained after the 3rd week were docosane (29.36%), nonacosane (10.99%), tetratetracontane (9.28%), and hexatriacontane (7.18%), which represented (56.81%) of the total peak area.

The presence of isolate A7 significantly altered the composition of hydrocarbons in the soil. The treated soil presented a relatively higher percentage of certain hydrocarbons, indicating enhanced degradation of the crude oil components. For example, hexacosane was not detected in treated soil after the second and third weeks. Additionally, the peak area of nonacosane declined from 3.92 to 0.33% over 3 weeks in the control soil, whereas in the treated soil it increased from 7.08 to 10.99%. Both hexacosane and nonacosane are long-chain alkanes identified in other studies involving the biodegradation of crude oil (Płaza et al. 2008; Koh and Khor 2023). Furthermore, certain compounds that are part of crude oil disappeared—or were significantly reduced—in treated soil. For example, hexadecane, a type of straight-chain alkanes, is one of the first alkanes to be degraded by microorganisms and was not detected in treated soil samples (Noveiri et al. 2024).

Many authors have investigated bioaugmentation for crude oil removal. Popoola et al. (2022) investigated the influence of process parameters on the biodegradation effectiveness of bacterial isolates in crude oil-polluted soil in Nigeria. The experiment was conducted over 60 days, using bacteria isolated from a native crude oil-polluted site. This study revealed that biostimulation significantly enhanced the degradation of TPH, achieving a degradation efficiency of up to 93.75% under optimal conditions. Additionally, Muthukumar et al. (2023) employed a bacterial consortium consisting of multiple *Pseudomonas* strains known for their hydrocarbon-degrading capabilities which demonstrated high efficiency in degrading crude oil under optimized conditions. Furthermore, Baoune et al. (2019) evaluated the performance of *Streptomyces* sp. Hlh1 in removing crude petroleum from contaminated soils. The strain was able to degrade total petroleum hydrocarbons (TPH), n-alkanes, and aromatic hydrocarbons, and—in non-sterilized soil—the maximum TPH removal reached 55% at an initial concentration of 2%.

### Identification of actinobacterial isolates

**Fig. 4:**
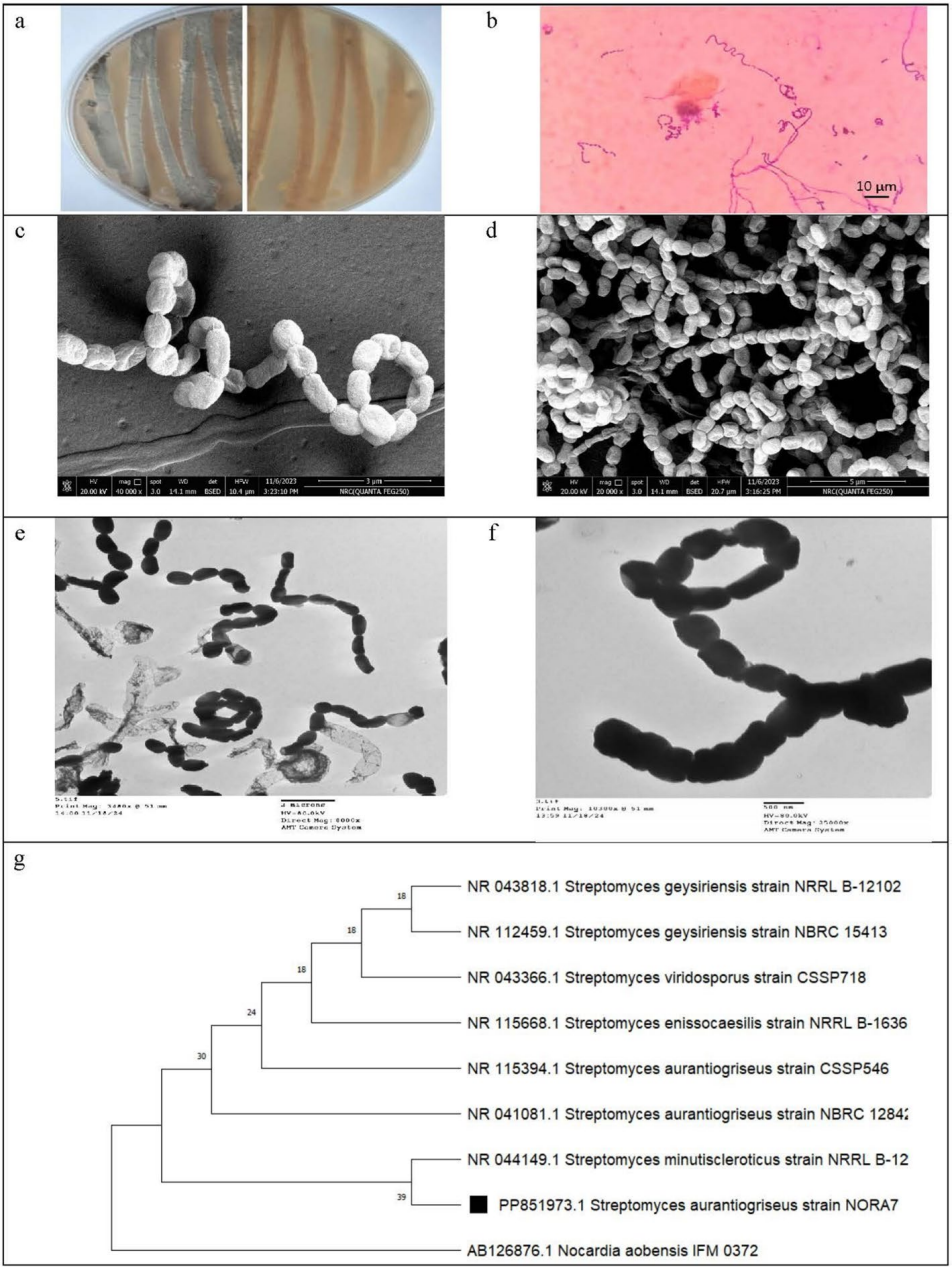
Morphological and cultural characteristics, and phylogenetic tree of *Streptomyces aurantiogriseus* strain NORA7 (isolate A7). **a** Culture on starch nitrate agar (forward and reverse), **b** coverslip culture stained with Gram stain, **c** SEM image (20.000 ×), **d** SEM image (40.000 ×) showing an open spiral spore chain with an elongated smooth surface of spores, **e** TEM image (8000 ×), **f** TEM image (24.000 ×) showing a smooth surface of spores, and **g** phylogenetic tree based on 16S rRNA sequences showing the relationship between *Streptomyces aurantiogriseus* strain NORA7 and closely related strains found in the GenBank database obtained via the neighbor-joining (NJ) method. The tree was constructed with Mega11, and *Nocardia aebensis* strain IFM 0372 was used as an outgroup

The isolated actinobacteria were identified on the basis of their morphological and biochemical characteristics. All the isolates belong to the genus *Streptomyces*, grey series, as they are aerobic, Gram-positive, non-acid-fast bacteria and form branched substrate and aerial mycelia. Some isolates could produce brown diffusible pigments on certain media. The spore chain arrangement was mono-verticillate except for isolates A11, A13, and B2 which are bi-verticillate. The spore chain morphology is either spiral or rectus flexibilis. The most effective isolate for crude oil biodegradation (isolate A7) formed long open spiral spore chains with smooth spores (Fig. 4a–f). Almost every tested C source supported the growth of nearly all the isolates. The studied actinobacteria could thrive in 2.5% NaCl, while some could thrive in 5%. All the examined isolates were sensitive to 50 µg/mL streptomycin and could grow on Czapeks agar and at pH values ranging from 5 to 9. None of the tested isolates exhibited antifungal activity against *A. flavus* or *P. chrysogenum*, but isolates B1, B2, B3, B5 and C2 exhibited antimicrobial activity against *C. albicans*. On the other hand, all the isolates, except isolates B3 and B5, had antibacterial effects on *B. subtilis* and/or *K. pneumonia*. More details on the phenotypic identification results are documented in Supplementary Tables S11–S15 and some of the performed tests are shown in Supplementary Figure S5.

Previous reports on hydrocarbon-degrading actinobacteria included *Arthrobacter*, *Gordonia*, *Micromonospora*, *Mycobacterium*, *Nocardia*, *Rhodococcus*, and most commonly *Streptomyces* (Iwahori et al. 1995; George et al. 2011; Mona Othman 2020; Lee and Kim 2022). Bacterial genera belonging to bacterial phyla other than Actinomycetota have also been examined for their ability to degrade crude oil, including *Acetobacter*, *Acinetobacter*, *Bacillus licheniformis*, *Pseudomonas aeruginosa*, and *Yarrowia lipolytica* (Muthukumar et al. 2022; Ferreira et al. 2023; Srimathi et al. 2024).

Agarose gel electrophoresis of the amplified DNA of the most bioactive isolate (A7) was performed, and an image of the purified DNA band on the gel is shown in Supplementary Figure S6. Phylogenetic analysis based on sequence analysis of the amplification product of 16S rRNA matches its phenotypic and biochemical identification. It showed 99.49% identity with *S. viridosporus* CSSP718, *S. geysiriensis* NBRC 15413 and NRRL B-12102. It also showed 99.41% identity with *S. eneissocaesilis* NRRL B-16365, 99.16% identity with *S. aurantiogriseus* NBRC 12842, and 99.07% with *S. aurantiogriseus* CSSP546 (Supplementary Table S16). Phylogenetic analysis grouped isolate A7 with *S. minutiscleroticus* NRRL B-12202 in one clade, with 99.94% sequence identity (Fig. 4g). Considering its cultural, morphological, and biochemical features, *S. aurantiogriseus* stands out as the species most closely aligned with isolate A7. Therefore, we recorded isolate A7 as *S. aurantiogriseus* strain NORA7 in the GenBank and the assigned accession No. was PP851973.

The phylogenetic tree based on the 16S rRNA gene sequence indicated that *S. aurantiogriseus* NORA7 and the closely related *Streptomyces* strains deposited in the NCBI database were separated into two major clades, and the tested strain was grouped with *S. viridosporus* CSSP718*, S. eneissocaesilis* NRRL B-16365, *S. geysiriensis* NBRC 15413 and *S. minutiscleroticus* NRRL B-12202 in one clade.

Figure 4f illustrates the genetic distance between *S. aurantiogriseus* NORA7 and its phylogenetically closest species deposited in the NCBI database. The tree was established using BLAST pairwise neighbor joining alignments.

*S. aurantiogriseus* NORA7 was deposited at the Egyptian Microbial Culture Collection (EMCC) as *S. aurantiogriseus* strain NORA7 EMCC 28565.

## Conclusion

Crude oil pollution demands sustainable and effective remediation strategies. Our study confirms the significant potential of biodegradation by *Streptomyces* species as an eco-friendly solution. We successfully isolated *Streptomyces aurantiogriseus* strain NORA7(EMCC 28565) which demonstrated robust crude oil degradation capability, initially achieving 66.28% degradation, which was further optimized by applying Plackett–Burman design and Response Surface Methodology (RSM). The Central Composite Design (CCD) model effectively predicted the optimal conditions, which were determined to be a 3% crude oil concentration, a 0.15 g/L yeast extract concentration, and a 25 mm agar plug diameter of the actinobacterium culture (inoculum size). Under these optimized conditions, our experimental validation revealed 70% removal of crude oil, which aligns closely with the model's predicted percentage of 74.43%. This small difference between the predicted and experimental values strongly affirms the efficacy of our developed models in accurately predicting the impact of *Streptomyces aurantiogriseus* strain NORA7. Gas chromatography further revealed the effective degradation of key long-chain hydrocarbons such as docosane, nonadecane, pentacosane, and 7-methylpentadecane. Crucially, pot-scale experiments validated these findings for ex situ soil remediation with a remarkable 92% crude oil removal in soil treated with the actinobacterial suspension. This unequivocally proves the strain's efficacy in real-world applications. These results highlight the potential of *Streptomyces aurantiogriseus* NORA7 as a potent bioagent for environmental remediation, offering a viable, eco-friendly approach for restoring oil-polluted ecosystems and mitigating the long-term environmental impact of crude oil contamination.

## Supplementary Information

Below is the link to the electronic supplementary material.

Supplementary file1 (DOCX 147 KB)

Supplementary file2 (DOCX 1042 KB)

## Data Availability

Data generated or analyzed during this study are presented in this article with its supplementary information files (1 and 2). The isolate's partial sequence of 16S rRNA gene was submitted to and is available for free download from NCBI GenBank and assigned an accession number (PP851973) using the BLASTN algorithm after comparing it to the sequences found in the National Center for Biotechnology Information (NCBI) database (https://www.ncbi.nlm.nih.gov/nuccore/PP851973).
