# Supplementary material for: Statistical optimization of crude oil bioremediation using Streptomyces aurantiogriseus isolated from Egypt’s Western Desert
Source: Biodegradation. 2025 Jul 25;36(4):68. doi: 10.1007/s10532-025-10154-0 (PMC12296995; doi:10.1007/s10532-025-10154-0)
Supplement: Supplementary file 1 — Supplementary file1 (DOCX 147 KB) [file 10532_2025_10154_MOESM1_ESM.docx]

**Article title:** Statistical optimization of crude oil bioremediation using *Streptomyces aurantiogriseus* isolated from Egypt's Western Desert

**Journal title:** Biodegradation

**Authors:** Sahar Y. Ibrahim^a,^*, Eman A. Abdelhamid^a,^*, Ali M. El-Hagrassi^b^, Noha M. Kamal^a,^*

**Affiliations:**

*^a^ Botany Department, Faculty of Women for Arts, Science, and Education, Ain Shams University, Cairo, Egypt. Postal code 11757*

*^b^ Department of Phytochemistry and Plant Systematics, Pharmaceutical Industries Research Institute, National Research Centre, 33 EL Buhouth St., Dokki, Giza 12622, Egypt*

***Corresponding authors:** Sahar Y. Ibrahim (E-mail: [sahar_moussa@women.asu.edu.eg](mailto:sahar_moussa@women.asu.edu.eg))

Noha M. Kamal (E-mail: [noha.ghanem@women.asu.edu.eg](mailto:noha.ghanem@women.asu.edu.eg))

Eman A. Abdelhamid (E-mail: eman.ahmed@women.asu.edu.eg)

**Supplementary Tables**

**Table S1** Description and locations of the collected polluted soil samples

| Feature | Soil sample | | |
| --- | --- | --- | --- |
|  | S-CRUDE | S-MOTOR | S-PEST |
| Description | A refinery crude oil-polluted soil from the petroleum extraction site, Marsa Matrouh Governorate, Western Desert. | A motor oil-polluted soil from an automobile repair workshop in Al Qalyubia Governorate. | A pesticide-polluted soil from agricultural soil in Al Qalyubia Governorate. |
| Latitude | 30.794104393790718 | 30.2255680 | 30.233330 |
| Longitude | 27.10830059088275 | 31.3298780 | 31.323147 |

**Table S2** The experimental range and variables used in the PB Design

| Name | Unit | Type | Low (-1) | High (+1) |
| --- | --- | --- | --- | --- |
| Concentration of hydrocarbon | % | Factor | 0.5 | 2 |
| Incubation time | Days | Factor | 5 | 10 |
| Agar plug diameter of isolate A7 | mm | Factor | 5 | 10 |
| Agar plug diameter of isolate A12 | mm | Factor | 5 | 10 |
| Agar plug diameter of isolate B1 | mm | Factor | 5 | 10 |
| Agar plug diameter of isolate A2 | mm | Factor | 5 | 10 |
| Temperature | °C | Factor | 25 | 40 |
| pH |  | Factor | 6 | 9 |
| Ferrous sulphate | g/L | Factor | 0.005 | 0.015 |
| Yeast extract | g/L | Factor | 0.05 | 0.15 |
| Glucose | g/L | Factor | 0.5 | 2 |
| Response (crude-oil removal) | % | Response | 32.2 | 60.4 |

# **Table S3** Physicochemical analysis of the polluted soil samples

| Parameter | S-CRUDE | S-MOTOR | S-PEST |
| --- | --- | --- | --- |
| PH | 8.34 | 8.27 | 8.87 |
| Conductivity, µS/cm at 25 °C | 104.8 | 588 | 140.9 |
| Organic matter, % | 6.72 | 15.36 | 13.95 |
| Texture | Silt loam | Sandy loam | Loamy sand |
| Sand, % | 46.126 | 55.892 | 84.644 |
| Silt, % | 50.029 | 42.697 | 14.603 |
| Clay, % | 3.845 | 1.411 | 0.753 |
| F^-^, ppm | 1.8 | 1.5 | 1.6 |
| Cl^-^, ppm | 11.5 | 320 | 24.1 |
| NO_2_^-^, ppm | ND | ND | ND |
| NO_3_^-^, ppm | ND | 16.3 | ND |
| PO_4_^-3^, ppm | ND | ND | ND |
| SO_4_^-2^, ppm | 8.4 | 229.5 | 16.8 |
| K, mg/Kg | 29.93 | 250.55 | 31.70 |
| Cu, mg/Kg | 0.299 | 0.289 | 0.390 |
| Cd, mg/Kg | ND | ND | ND |
| Pb, mg/Kg | ND | ND | ND |
| Cr, mg/Kg | ND | ND | ND |
| Zn, mg/Kg | 5.79 | 1.25 | 0.49 |

ND: not detected

**Table S4** Compounds detected in the GC spectra of the polluted soil samples

| No. | Compound | MW (g/mol) | MF | Area (%) | | | R_t_ (min) | | |
| --- | --- | --- | --- | --- | --- | --- | --- | --- | --- |
|  |  |  |  | S-CRUDE | S-MOTOR | S-PEST | S-CRUDE | S-MOTOR | S-PEST |
| 1 | 1,2-Benzisothiazole, 3-(hexahydro-1H-azepin-1-yl)-, 1,1-dioxide | 264.4 | C_13_H_16_N_2_O_2_S | 1.09 | ND | ND | 14.7 | ND | ND |
| 2 | 11,13-Dimethyl-12-tetradecen-1-ol acetate | 282.5 | C_18_H_34_O_2_ | 0.69 | ND | ND | 18.1 | ND | ND |
| 3 | 1,2-Benzene dicarboxylic acid | 390 | C_24_H_38_O_4_ | ND | ND | 9.35 | ND | ND | 36.71 |
| 4 | 15-Methyl tricyclo [6.5.2(13,14).0(7,15)] pentadeca -1,3,5,7,9,1 1,13-heptene | 206 | C_16_H_14_ | ND | ND | 1.94 | ND | ND | 17.53 |
| 5 | 1-Octadecene | 252.5 | C_18_H_36_ | 2.67 | ND | ND | 12.80 | ND | ND |
| 6 | 2,5-Cyclohexadien-1-One, 2,6-bis(1,1-dimethylethyl)-4 methylene | 218 | C_15_H_22_O | ND | ND | 3.45 | ND | ND | 16.65 |
| 7 | 2,6,10,14-Tetramethyl-7-(3-methylpent-4-enylidene) pentadecane | 348.6 | C_25_H_48_ | 2.29 | ND | ND | 13.20 | ND | ND |
| 8 | 2,6,10-Trimethyl, tetradecane | 240 | C_17_H_36_ | ND | ND | 2.87 | ND | ND | 19.96 |
| 9 | 2,6,10-Trimethyltridecane | 226.4 | C_16_H_34_ | 0.20 | ND | ND | 9.00 | ND | ND |
| 10 | 2-Acetyl-3-(2-cinnamido) ethyl-7-methoxyindole | 362 | C_22_H_22_N_2_O_3_ | ND | ND | 1.20 | ND | ND | 33.27 |
| 11 | 2-Hexadecanol | 242 | C_16_H_34_O | ND | ND | 1.86 | ND | ND | 32.62 |
| 12 | 2-Methyl-1-hexadecanol | 256 | C_17_H_36_O | ND | ND | 16.17 | ND | ND | 18.45 |
| 13 | 3,5-Pyridine-dicarbonitrile,2-amino-6-(4-bromophenoxy) | 314 | C_13_H_7_BrN_4_O | ND | ND | 1.93 | ND | ND | 27.07 |
| 14 | 3,7,11-Trimethyl-1-dodecanol | 228 | C_15_H_32_O | ND | ND | 7.64 | ND | ND | 18.22 |
| 15 | 1,2,4,5-Tetraethyl, cyclohexane | 196.4 | C_14_H_28_ | ND | -0.04 | ND | ND | 9.05 | ND |
| 16 | Cyclotetradecane | 196.4 | C_14_H_28_ | ND | 0.31 | ND | ND | 12.6 | ND |
| 17 | 1,7,11-Trimethyl-4-(1-methylethyl)-cyclotetradecane | 280.5 | C_20_H_40_ | ND | 0.19 | ND | ND | 12.8 | ND |
| 18 | Cyclotriacontane | 420.8 | C_30_H_60_ | ND | 0.05 | ND | ND | 9.58 | ND |
| 19 | 3,8-Dimethyl, decane | 170.3 | C_12_H_26_ | ND | 0.94 | ND | ND | 20.7 | ND |
| 20 | Diisooctyl phthalate | 390.6 | C_24_H_38_O_4_ | 0.44 | ND | ND | 9.55 | ND | ND |
| 21 | Docosane | 310.6 | C_22_H_46_ | 5.33 | ND | ND | 12.3 | ND | ND |
| 22 | 4,6-Dimethyl, dodecane | 198.4 | C_14_H_30_ | ND | 0.08 | ND | ND | 10.7 | ND |
| 23 | Dodecyl acrylate | 240 | C_15_H_28_O_2_ | ND | ND | 14.42 | ND | ND | 21.67 |
| 24 | Dotriacontane | 450 | C_32_H_66_ | ND | ND | 6.37 | ND | ND | 17.62 |
| 25 | Dotriacontyl pentafluoropropionate | 612.9 | C_33_H_65_F_5_O_2_ | -0.12 | ND | ND | 8.02 | ND | ND |
| 26 | Eicosane | 282.5 | C_20_H_42_ | 0.11 | ND | ND | 8.30 | ND | ND |
| 27 | 2-(Octadecyloxy), ethanol | 314.5 | C_20_H_42_O_2_ | ND | 0.06 | ND | ND | 10.20 | ND |
| 28 | Heneicosane | 296.6 | C_21_H_44_ | 0.84 | ND | ND | 18.00 | ND | ND |
| 29 | Hentriacontane | 436.8 | C_31_H_64_ | ND | 5.03 | ND | ND | 14.90 | ND |
| 30 | Heptacosane | 380.7 | C_27_H_56_ | 15.50 | 7.30 | ND | 13.40 | 13.40 | ND |
| 31 | Heptadecane | 240.5 | C_17_H_36_ | 0.12 | ND | ND | 8.60 | ND | ND |
| 32 | Hexacosane | 366.7 | C_26_H_54_ | 5.50 | 0.82 | ND | 12.50 | 12.50 | ND |
| 33 | Hexadecane | 226.4 | C_16_H_34_ | 6.37 | 0.25 | ND | 12.90 | 12.90 | ND |
| 34 | 2,6,10,14-Tetramethyl, hexadecane | 348.6 | C_25_H_48_ | 7.75 | ND | ND | 17.8 | ND | ND |
| 35 | Hexadecyl ester, trichloroacetic acid | 386 | C_18_H_33_Cl_3_O_2_ | ND | ND | 2.32 | ND | ND | 23.04 |
| 36 | Hexatriacontane | 507 | C_36_H_74_ | 4.15 | ND | ND | 15.50 | ND | ND |
| 37 | Isochiapin B | 346 | C_19_H_22_O_6_ | ND | ND | 30.49 | ND | ND | 27.38 |
| 38 | Nonacosane | 408.8 | C_29_H_60_ | 5.81 | 0.25 | ND | 11.00 | 11.00 | ND |
| 39 | Nonadecane | 268.5 | C_19_H_40_ | 9.44 | 12.10 | ND | 14.80 | 15.10 | ND |
| 40 | Nonahexacontanoic acid | 999.8 | C_69_H_138_O_2_ | 3.23 | 0.14 | ND | 10.40 | 10.40 | ND |
| 41 | 4,5-Dimethyl, nonane | 156.3 | C_11_H_24_ | 0.15 | 13.70 | ND | 22.40 | 19.00 | ND |
| 42 | Octacosane | 394.8 | C_28_H_58_ | 6.71 | ND | ND | 15.70 | ND | ND |
| 43 | Octadecane | 254.5 | C_18_H_38_ | 9.10 | 0.68 | ND | 12.00 | 12.00 | ND |
| 44 | 1-Bromo, octadecane | 333.4 | C_18_H_37_Br | 0.06 | ND | ND | 10.80 | 0.00 | ND |
| 45 | 1-Chloro, octadecane | 288.9 | C_18_H_37_Cl | 0.61 | 11.60 | ND | 17.70 | 15.90 | ND |
| 46 | Octatriacontyl pentafluoropropionate | 697 | C_41_H_77_F_5_O_2_ | 7.59 | 0.57 | ND | 11.50 | 11.50 | ND |
| 47 | Pentadecane | 212.4 | C_15_H_32_ | 0.18 | ND | ND | 20.40 | ND | ND |
| 48 | Tetracosane | 338.7 | C_24_H_50_ | 0.89 | 0.07 | ND | 9.81 | 9.86 | ND |
| 49 | Tetradecane | 198.4 | C_14_H_30_ | 1.72 | 1.71 | ND | 14.2 | 14.20 | ND |
| 50 | 2-Hydroxy-, methyl ester, tetradecanoic acid | 258.4 | C_15_H_30_O_3_ | 0.31 | ND | ND | 9.20 | ND | ND |
| 51 | Tetratriacontane | 478.9 | C_34_H_70_ | 3.21 | 7.56 | ND | 18.60 | 19.30 | ND |
| 52 | Triacontane | 422.8 | C_30_H_62_ | 1.93 | ND | ND | 16.40 | ND | ND |
| 53 | Tricosane | 324.6 | C_23_H_48_ | 8.13 | 12.90 | ND | 13.80 | 13.80 | ND |
| 54 | Z-14-Nonacosane | 406.8 | C_29_H_58_ | 11.80 | ND | ND | 17.10 | ND | ND |

R_t_, retention time; MF, molecular formula; MW, molecular weight; ND, not detected.

**Table S5** Compounds detected in the GC spectrum of the untreated crude oil only

| No. | Compound | Area (%) | R_t_ (min) | MW (g/mol) | MF |
| --- | --- | --- | --- | --- | --- |
| 1 | 4-Chloro, octane | 0.52 | 22.53 | 148 | C_8_H_17_Cl |
| 2 | (5,10,15,20-Tetraphenyl- [2 (2)h1] prophyrinato) zinx(ii) | 0.73 | 35.68 | 679 | C_44_H_27_DN_4_Zn |
| 3 | [4,4-2H2]-1-Bromopent-2-yne | 0.56 | 29.45 | 146 | C_5_H_5_D_2_Br |
| 4 | 1,2-Nonadecanediol | 1.96 | 38.99 | 300 | C_19_H_40_O_2_ |
| 5 | 10-Undecenoic acid, octyl ester | 1.80 | 45.33 | 88 | C_19_H_36_O_2_ |
| 6 | 11-(1-Ethylpropyl),heneicosane  Another name: 11-Pentan-3-ylhenicosane | 4.49 | 44.59 | 366 | C_26_H_54_ |
| 7 | 1-Chloro-7-heptadecene | 0.48 | 37.48 | 272 | C_17_H_33_Cl |
| 8 | 1-Dodecene | 10.15 | 29.76 | 168 | C_12_H_24_ |
| 9 | 1H-Cyclopropa [3,4] benz[1,2e] azulene-4a,5,7b,9,9a(1ah)-pentol,3-[(acetyloxy)-methyl]-1b,4,5,7a,8,9-hexahydro-1,1,6,8-tetramethyl,9,9-a-diacetate, [1aR(1aà,1bá,4aá,5à,7aà,7bà,8à,9á,9aà)] | 0.40 | 47.23 | 492 | C_26_H_36_O_9_ |
| 10 | 4-Benzyloxy-4- [2,2, -dimethyl-4-dioxolanyl], butylaldehyde | 0.64 | 32.73 | 278 | C_16_H_22_O_4_ |
| 11 | 6-Aminohexanamide, N-methyl-N-[4-(1-pyrrolidinyl)-2-butynyl]-N-'t-butyloxycarbonyl | 0.47 | 26.54 | 365 | C_20_H_35_N_3_O_3_ |
| 12 | 8,9,13-Trihydroxy-, methyl ester docosanoic acid, another name: Methyl 8,9,13-trihydroxydocosanoate | 0.53 | 39.08 | 402 | C_23_H_46_O_5_ |
| 13 | Allopumiliotoxin | 0.36 | 29.08 | 339 | C_19_H_33_NO_4_ |
| 14 | Butanedioic acid monoamidemonohydrazide, N-(4-fluorophenyl)-N''-(4-nitrobenzylideno) | 0.27 | 36.63 | 358 | C_17_H_15_FN_4_O_4_ |
| 15 | Colchifoleine | 0.31 | 48.64 | 401 | C_21_H_23_NO_7_ |
| 16 | D5-benzoyl-val(O-D5-benzoyl) thr-bro-ome | 0.33 | 35.19 | 599 | C_34_H_27_D_10_N_3_O_7_ |
| 17 | I-Propyl-11,12-methylene-octadecanoate | 0.32 | 33.05 | 338 | C_22_H_42_O_2_ |
| 18 | N, N'-Pentamethylenebis [s-3-aminopropyl thiosulfuric acid] | 0.27 | 48.03 | 410 | C_11_H_26_N_2_O_6_S_4_ |
| 19 | 2-[5-(2-Hydroxypropyl) tetrahydrofuran-2-yl], -1-[5-(1-methoxy-1-oxopropan-2-yl) tetrahydro furan-2-yl] propan-2-yl ester, propanoic acid | 0.41 | 40.05 | 400 | C_21_H_36_O_7_ |
| 20 | Tetrabutyltitanate | 0.46 | 38.19 | 340 | C_16_H_36_O_4_Ti |
| 21 | Tetrahydroaraucarolone | 0.33 | 46.60 | 338 | C_20_H_34_O_4_ |
| 22 | Tetratetracontane | 0.35 | 37.10 | 618 | C_44_H_90_ |
| 23 | N-[3-[[3-[[(3R)-1-(cyclohexylmethyl)piperidin-3-yl]methylamino]-3-oxopropyl]amino]-3-oxopropyl]-3,3,3-triphenylpropanamide  Another name: Villalstonintriol | 0.41 | 37.23 | 636 | C_40_H_52_N_4_O_3_ |

R_t_, retention time; MF, molecular formula; MW, molecular weight.

**Table S6** Compounds detected in the GC spectra of untreated crude oil and all treatments

| No. | Compound | MW (g/mol) | MF | Area (%) | | | | | R_t_ (min) | | | | |
| --- | --- | --- | --- | --- | --- | --- | --- | --- | --- | --- | --- | --- | --- |
|  |  |  |  | Control | A2 | A7 | A12 | B1 | Control | A2 | A7 | A12 | B1 |
| 1 | 1-Tetradecanol | 214 | C_14_H_30_O | 6.06 | 2.32 | 0.53 | 2.77 | 0.46 | 39.62 | 26.26 | 24.13 | 22.51 | 37.21 |
| 2 | 2,2,4,9,11,11-Hexamethyl, dodecane | 254 | C_18_H_38_ | 10.86 | 3.95 | 3.02 | 3.84 | 2.72 | 28.9 | 22.52 | 30.63 | 33.04 | 26.25 |
| 3 | 2,6,10-Trimethyl, tetradecane | 240 | C_17_H_36_ | 3.98 | 6.78 | 0.93 | 21.06 | 1.51 | 27.47 | 23.72 | 26.58 | 29.74 | 26.56 |
| 4 | 2-Myristynoyl pantetheine | 484 | C_25_H_44_N_2_O_5_S | 1.13 | 0.51 | 0.56 | 0.40 | 0.53 | 31.27 | 40.04 | 30.75 | 40.04 | 47.01 |
| 5 | Docosane | 310 | C_22_H_46_ | 23.60 | 3.24 | 10.35 | 9.67 | 10.31 | 28.46 | 31.26 | 24.49 | 21.79 | 26.44 |
| 6 | Hexadecane | 226 | C_16_H_34_ | 1.84 | 19.87 | 0.25 | 7.25 | 6.24 | 25.07 | 29.78 | 24.31 | 27.46 | 23.98 |

R_t_, retention time; MF, molecular formula; MW, molecular weight.

**Table S7** Compounds detected in the GC spectra of untreated crude oil and some treatments

| No. | Compound | MW (g/mol) | MF | Area (%) | | | | | R_t_ (min) | | | | |
| --- | --- | --- | --- | --- | --- | --- | --- | --- | --- | --- | --- | --- | --- |
|  |  |  |  | Control | A2 | A7 | A12 | B1 | Control | A2 | A7 | A12 | B1 |
| 1 | 4-[3(3,4-Dimethoxy phenyl)-1-oxo -1-1-thiophene-2-yl-1,2,3,4,5,11-hexahydro-dibenzo [ b, e] [1,4] diazepin-10-yl] -4-oxo-butyric acid | 532 | C_29_H_28_N_2_O_6_S | 0.38 | ND | ND | 0.34 | ND | 48.47 | ND | ND | 38.18 | ND |
| 2 | 9-Octadecenoic acid (Z) | 282 | C_18_H_34_O_2_ | 0.34 | 0.61 | ND | ND | 14.89 | 33.19 | 37.47 | ND | ND | 39.20 |
| 3 | 1,1-Bis(dodecyloxy), hexadecane | 594 | C_40_H_82_O_2_ | 0.35 | 0.84 | ND | ND | 0.27 | 44.38 | 37.20 | ND | ND | 32.51 |
| 4 | 9-Hexyl,heptadecane | 324 | C_23_H_48_ | 0.77 | 2.30 | ND | ND | 1.17 | 33.65 | 33.36 | ND | ND | 30.63 |
| 5 | Heneicosane | 296 | C_21_H_44_ | 6.41 | ND | ND | ND | 3.69 | 37.82 | ND | ND | ND | 37.84 |
| 6 | Docosyltrichlorosilane | 442 | C_22_H_45_Cl_3_Si | 17.76 | 5.76 | ND | 2.67 | ND | 23.99 | 35.95 | ND | 35.17 | ND |

R_t_, retention time; MF, molecular formula; MW, molecular weight.

**Table S8** Compounds detected in the GC-MS spectra of treated crude oil only

| No. | Compound | MW (g/mol) | MF | Area (%) | | | | R_t_ (min) | | | |
| --- | --- | --- | --- | --- | --- | --- | --- | --- | --- | --- | --- |
|  |  |  |  | A2 | A7 | A12 | B1 | A2 | A7 | A12 | B1 |
| 1 | (Ethenyloxy)- isooctane  Another name: 6-Methylheptyl vinyl ether | 156 | C_10_H_20_O | 0.46 | ND | ND | ND | 24.13 | ND | ND | ND |
| 2 | (Z, Z)-9,12-Octadecadienoic acid, methyl ester, another name: Linoleic acid, methyl ester | 294 | C_19_H_34_O_2_ | 0.55 | ND | ND | ND | ND | 27.63 | ND | ND |
| 3 | 1-(1-Methylethyl)-2-nonyl, cyclopropane | 210 | C_15_H_30_ | ND | 0.40 | ND | ND | ND | 29.08 | ND | ND |
| 4 | 1-(Ethenyloxy)- octadecane  Another name: Octadecyl vinyl ether | 296 | C_20_H_40_O | 3.54 | 0.27 | ND | ND | 35.36 | 35.35 | ND | ND |
| 5 | 1,1-Dimethoxy, octadecane | 314 | C_20_H_42_O_2_ | ND | ND | 0.48 | 2.82 | ND | ND | 35.68 | 35.47 |
| 6 | 1,1'-Dodecylidene -bis [4-methyl cyclohexane] | 362 | C_26_H_50_ | ND | ND | ND | 0.31 | ND | ND | ND | 37.47 |
| 7 | 1,1'-Oxybis, decane | 298 | C_20_H_42_O | ND | 1.93 | ND | ND | ND | 24.00 | ND | ND |
| 8 | 1,2-Benzenedicarboxylic acid, bis(2-ethylhexyl) ester  Another name: Diethylhexylphthalate | 390 | C_24_H_38_O_4_ | ND | ND | 1.43 | ND | ND | ND | 45.31 | ND |
| 9 | 1,2-Benzenedicarboxylic acid, dioctyl ester | 390 | C_24_H_38_O_4_ | ND | ND | ND | 0.83 | ND | ND | ND | 45.33 |
| 10 | 1,2-Dibromo-2-methyl, undecane | 326 | C_12_H_24_Br_2_ | 0.45 | ND | ND | ND | 35.67 | ND | ND | ND |
| 11 | 1,4-Butanediamine | 88 | C_4_H_12_N_2_ | 0.49 | ND | ND | ND | 35.67 | ND | ND | ND |
| 12 | 1,4-Dimethyl-2-octadecyl, cyclohexane | 364 | C_26_H_52_ | ND | 0.21 | ND | ND | 39.08 | ND | ND | ND |
| 13 | 11-Octadecenal | 266 | C_18_H_34_O | ND | ND | 0.72 | ND | ND | ND | 35.35 | ND |
| 14 | 13-Heptadecyn-1-ol | 252 | C_17_H_32_O | ND | ND | 1.74 | ND | ND | ND | 35.35 | ND |
| 15 | 17-Pentatriacontene | 490 | C_35_H_70_ | ND | 1.68 | 0.96 | ND | ND | 49.02 | 32.71 | ND |
| 16 | 1-Bromo-5-heptadecene | 316 | C_17_H_33_Br | ND | 0.45 | ND | ND | ND | 38.96 | ND | ND |
| 17 | 1H-Thieno[3,4d] imidazole-4-pentanoic acid, hexahydro-2-oxo,5-oxide, [3as-(3aà,4á,6aà)] | 260 | C_10_H_16_N_2_O_4_S | ND | ND | ND | 0.53 | ND | ND | ND | 30.74 |
| 18 | 1-Oxaspiro [4.4] nonan-4-one, 2-isopropyl | 182 | C_11_H_18_O_2_ | ND | 0.83 | ND | ND | ND | 28.91 | ND | ND |
| 19 | 2-(3-Butynyloxy) tetrahydro, 2H-pyran | 154 | C_9_H_14_O_2_ | ND | ND | 1.18 | 0.87 | ND | ND | 46.11 | 28.88 |
| 20 | 2-(Octadecyloxy)ethanol | 314 | C_20_H_42_O_2_ | ND | ND | ND | 0.25 | ND | ND | ND | 37.10 |
| 21 | 2,2-Dideutero octadecanal | 268 | C_18_H_34_D_2_O | 0.32 | ND | 0.52 | ND | 26.73 | ND | 33.06 | ND |
| 22 | 2,3-Dihydroxypropylester, hexadecanoic acid | 330 | C_19_H_38_O_4_ | ND | ND | ND | 5.75 | ND | ND | ND | 35.37 |
| 23 | 2,6-Dimethyl, 1-heptanol | 144 | C_9_H_20_O | 0.94 | ND | ND | ND | 21.80 | ND | ND | ND |
| 24 | 2,6-Dimethyldecane | 170 | C_12_H_26_ | 2.60 | ND | ND | ND | 23.99 | ND | ND | ND |
| 25 | 2-Butyloxycarbonyloxy-1,1,10-trimethyl-6,9-epidioxydecalin | 326 | C_18_H_30_O_5_ | ND | ND | 0.73 | ND | ND | ND | 30.62 | ND |
| 26 | 2-Cyclohexyleicosane | 364 | C_26_H_52_ | 1.43 | ND | 0.90 | ND | 28.91 | ND | 35.46 | ND |
| 27 | 2-D-2-Pentadecyl,1,3-dioxolane | 284 | C_18_H_35_DO_2_ | ND | ND | ND | 0.68 | ND | ND | ND | 5.08 |
| 28 | 2-Ethyl-1-decanol | 186 | C_12_H_26_O | 5.30 | ND | ND | ND | 27.48 | ND | ND | ND |
| 29 | 2-Methyl, pentadecane | 226 | C_16_H_34_ | ND | ND | ND | 0.58 | ND | ND | ND | 31.10 |
| 30 | 2-Methyl, undecane | 170 | C_12_H_26_ | ND | 3.67 | ND | ND | ND | 37.83 | ND | ND |
| 31 | 2-Methyl-1-hexadecanol | 256 | C_17_H_36_O | 0.82 | ND | 0.69 | 0.79 | 29.08 | ND | 39.06 | 29.08 |
| 32 | 2-Methyl-Z-4-tetradecene | 210 | C_15_H_30_ | 1.04 | ND | ND | ND | 31.11 | ND | ND | ND |
| 33 | 2-Octadecyloxy-1,1,2,2-tetradeutero, ethanol | 314 | C_20_H_38_D_4_O_2_ | ND | 0.34 | ND | ND | ND | 37.20 | ND | ND |
| 34 | 2¬-Methyl¬, dodecyl ester, 2-¬propenoic acid | 254 | C_16_H_30_O_2_ | 1.79 | ND | 0.81 | ND | 26.44 | ND | 26.43 | ND |
| 35 | 1,1,1,2-Tetrafluoro-, (Z) 2-tridecene | 254 | C_13_H_22_F_4_ | ND | 2.50 | ND | ND | ND | 22.54 | ND | ND |
| 36 | 3-(2,3,6-Trimethyl-1,4-dioxaspiro [4.4] non-7-yl), methyl ester, propanoic acid | 256 | C_14_H_24_O_4_ | 0.32 | ND | ND | ND | 31.69 | ND | ND | ND |
| 37 | 3,4-Dimethyl, heptane | 128 | C_9_H_20_ | ND | ND | 1.49 | ND | ND | ND | 23.97 | ND |
| 38 | 3,4-Dimethyl, octane | 142 | C_10_H_22_ | ND | 5.96 | ND | ND | ND | 34.00 | ND | ND |
| 39 | 3,7,11-Trimethyl,1-dodecanol | 228 | C_15_H_32_O | 16.05 | ND | ND | ND | 32.00 | ND | ND | ND |
| 40 | 3-Ethyl-5-(2-ethylbutyl), octadecane | 366 | C_26_H_54_ | ND | ND | ND | 1.00 | ND | ND | ND | 45.12 |
| 41 | 3-Hydroxy, dodecanoic acid | 216 | C_12_H_24_O_3_ | ND | 0.82 | ND | ND | ND | 26.44 | ND | ND |
| 42 | 3-Methylene-7,11,15-trimethylhexadecadiene-4-hydroperoxide | 276 | C_20_H_36_ | ND | ND | 1.26 | ND | ND | ND | 5.07 | ND |
| 43 | 3-Trifluoroacetoxy, dodecane | 282 | C_14_H_25_F_3_O_2_ | 4.29 | ND | ND | ND | 25.07 | ND | ND | ND |
| 44 | 3-Trifluoroacetoxypenta, decane | 324 | C_17_H_3_1F_3_O_2_ | ND | ND | 3.49 | ND | ND | ND | 39.59 | ND |
| 45 | 4- [3(3,4-dimethoxy phenyl)-1-oxo -1-1-thiophen-2-yl-1,2,3,4,5,11-hexahydro-dibenzo [ b, e] [1,4] diazepin-10-yl] -4-oxo-butyric acid | 532 | C_29_H_28_N_2_O_6_S | ND | ND | 0.34 | ND | ND | ND | 38.18 | ND |
| 46 | 4,5,6,7-Tetrahydro-3-acetamido -n-allyl- benzothiophene-2-carboxamide | 278 | C_14_H_18_N_2_O_2_S | ND | 0.22 | ND | ND | ND | 35.46 | ND | ND |
| 47 | 4,5-Dimethyl, nonane | 156 | C_11_H_24_ | ND | 7.09 | ND | ND | ND | 25.10 | ND | ND |
| 48 | 4-Methoxy-2-methylbenzoic acid | 166 | C_9_H_10_O_3_ | 2.21 | 0.60 | ND | ND | 45.33 | 45.32 | ND | ND |
| 49 | 5,8-Diethyl, dodecane | 226 | C_16_H_34_ | 2.02 | 0.64 | 0.38 | 0.37 | 26.76 | 26.76 | 32.49 | 26.75 |
| 50 | 5-A-Methyl-3,8-dimethylene -2-oxo dodecahydrooxireno [2',3':6,7] naphtho [1,2-b] furan-6-yl 2-methyl-2-butenoate | 344 | C_20_H_24_O_5_ | 0.56 | ND | ND | ND | 35.47 | ND | ND | ND |
| 51 | 5-Butyl, hexadecane | 282 | C_20_H_42_ | ND | 0.19 | ND | ND | ND | 33.19 | ND | ND |
| 52 | 5-Methyl, octadecane | 268 | C_19_H_40_ | ND | ND | 0.82 | ND | ND | ND | 33.63 | ND |
| 53 | 5-Methyl, pentadecane | 226 | C_16_H_34_ | ND | 5.02 | ND | ND | ND | 35.96 | ND | ND |
| 54 | 7-Methyl, pentadecane | 226 | C_16_H_34_ | ND | 20.22 | ND | ND | ND | 29.8 | ND | ND |
| 55 | 7-Oxo-2-oxa-7-thiatri cyclo [4.4.0.0(3,8)] decan-4-ol | 188 | C_8_H_12_O_3S_ | 0.92 | ND | ND | ND | 38.97 | ND | ND | ND |
| 56 | 8-Methyl -7-(4-morpholinyl)-4,5,7,8-tetrahydro-8-ah [1,2,5] oxadiazolo [3,4-e] indol-8a-ol-6-oxide | 294 | C_13_H_18_N_4_O_4_ | ND | ND | 0.32 | ND | ND | ND | 29.43 | ND |
| 57 | 9-Dodecyltetradecahydro, phenanthrene | 360 | C_26_H_48_ | ND | 0.16 | ND | ND | ND | 48.01 | ND | ND |
| 58 | 9-Octadecen-12-ynoic acid, methyl ester | 292 | C_19_H_32_O_2_ | 0.32 | 0.51 | ND | 0.26 | 41.4 | 31.11 | ND | 48.64 |
| 59 | Aspartame | 294 | C_14_H_18_N_2_O_5_ | ND | ND | 0.17 | ND | ND | 25.69 | ND | ND |
| 60 | Ceanothine C | 470 | C_26_H_38_N_4_O_4_ | 0.81 | ND | 0.67 | ND | 30.64 | ND | 37.19 | ND |
| 61 | Dibutyl ester, nonanedioic acid | 300 | C_17_H_32_O_4_ | ND | ND | ND | 0.27 | ND | ND | ND | 35.77 |
| 62 | Eicosane | 282 | C_20_H_42_ | ND | ND | 5.75 | 5.22 | ND | ND | 35.92 | 35.96 |
| 63 | Ethyl-3-(a-chloro-4-cyclohexylbenzylidene)-4-(2,4-dinitrophenylhydrazono), butyrate | 514 | C_25_H_27_ClN_4_O_6_ | 0.39 | ND | ND | ND | 28.2 | ND | ND | ND |
| 64 | Heptadecane | 240 | C_17_H_36_ | ND | 3.3 | ND | 13.07 | ND | 39.63 | ND | 29.78 |
| 65 | Limonen-6-ol, pivalate | 236 | C_15_H_24_O_2_ | 0.48 | ND | ND | ND | 29.45 | ND | ND | ND |
| 66 | Nonacosane | 408 | C_29_H_60_ | ND | ND | ND | 1.17 | ND | 27.51 | ND | 38.95 |
| 67 | Nonadecane | 268 | C_19_H_40_ | ND | 10.04 | ND | ND | ND | 27.51 | ND | ND |
| 68 | Octadecane | 254 | C_18_H_38_ | ND | 0.62 | ND | 12.02 | ND | 21.81 | ND | 31.94 |
| 69 | Octadecanoic acid (other name: stearic acid) | 284 | C_18_H_36_O_2_ | ND | ND | ND | 1.60 | ND | ND | ND | 39.41 |
| 70 | Paromomycin | 615 | C_23_H_45_N_5_O_14_ | ND | ND | 0.29 | ND | ND | ND | 34.52 | ND |
| 71 | Pentacosane | 352 | C_25_H_52_ | ND | 12.73 | 17.60 | 6.47 | ND | 31.96 | 31.91 | 33.99 |
| 72 | Pentadecane | 212 | C_15_H_32_ | ND | ND | 8.53 | 2.65 | ND | ND | 25.05 | 25.07 |
| 73 | 2- [4, á, á.-Trimethyl-5-imidazolyl]-ethyl]-, diethyl(ester), propanedioic acid | 296 | C_15_H_24_N_2_O_4_ | ND | ND | ND | 0.38 | ND | ND | ND | 34.34 |
| 74 | Tetradecane | 198 | C_14_H_30_ | 5.40 | ND | ND | 0.31 | 33.99 | ND | ND | 22.52 |
| 75 | Tetraneurin-a-diol  Another name: Autumnolide | 280 | C_15_H_20_O_5_ | ND | 0.20 | ND | ND | ND | 37.47 | ND | ND |
| 76 | Tricosane | 324 | C_23_H_48_ | ND | 2.52 | ND | ND | ND | 41.35 | ND | ND |
| 77 | Tridecanol | 200 | C_13_H_28_O | ND | 1.05 | 1.25 | ND | ND | 26.26 | 26.53 | ND |
| 78 | Z-(13,14-Epoxy) tetradic-11-en-1-ol- acetate | 268 | C_16_H_28_O_3_ | 0.33 | ND | ND | ND | 25.68 | ND | ND | ND |

R_t_, retention time; MF, molecular formula; MW, molecular weight; ND, not detected.

**Table S9** Coefficients in terms of coded factors

| Factor | Coefficient Estimate | df | Standard Error | VIF |
| --- | --- | --- | --- | --- |
| Intercept | 46.62 | 1.00 | 0.09 |  |
| A-Hydrocarbon | 3.97 | 1.00 | 0.09 | 1.00 |
| B-Incubation time | 3.05 | 1.00 | 0.09 | 1.00 |
| C- Isolate A7 | 2.31 | 1.00 | 0.09 | 1.00 |
| E- Isolate B1 | -1.37 | 1.00 | 0.09 | 1.00 |
| F- Isolate A2 | 1.30 | 1.00 | 0.09 | 1.00 |
| G-Temperature | -2.18 | 1.00 | 0.09 | 1.00 |
| H-pH | 3.01 | 1.00 | 0.09 | 1.00 |
| J-Ferrous sulphate | 3.10 | 1.00 | 0.09 | 1.00 |
| K-Yeast extract | 3.85 | 1.00 | 0.09 | 1.00 |
| L-Glucose | -2.70 | 1.00 | 0.09 | 1.00 |

dF= Degree of freedom, VIF= variance inflation factor.

**Table S10** Compounds detected in the GC spectra of the control and treated soils during bioaugmentation experiment

| Identified compounds | MW | MF | Week 1 | | | | Week 2 | | | | Week 3 | | | |
| --- | --- | --- | --- | --- | --- | --- | --- | --- | --- | --- | --- | --- | --- | --- |
|  |  |  | Control soil | | Treated soil | | Control soil | | Treated soil | | Control soil | | Treated soil | |
|  |  |  | Area  (%) | R_t_  (min.) | Area (%) | R_t_  (min.) | Area  (%) | R_t_  (min.) | Area  (%) | R_t_  (min.) | Area  (%) | R_t_  (min.) | Area  (%) | R_t_  (min.) |
| (1-Hexyltetradecyl), benzene | 358 | C_26_H_46_ | 0.74 | 39.80 | ND | ND | ND | ND | ND | ND | ND | ND | ND | ND |
| [3-(2-Cyclohexylethyl)-6-cyclopentylhexyl], benzene | 340 | C₂₅H₄₀ | ND | ND | ND | ND | ND | ND | ND | ND | ND | ND | 1.35 | 39.90 |
| (1-Hexadecyl heptadecyl)- benzene | 540 | C_39_H_72_ | ND | ND | 0.47 | 43.52 | ND | ND | ND | ND | ND | ND | ND | ND |
| (1-Propylheptadecyl), benzene | 358 | C_26_H_46_ | ND | ND | ND | ND | 0.78 | 28.92 | ND | ND | ND | ND | ND | ND |
| (1R,3S)-3-Hydroxymeth yl-1,2,2-trimethylcyclo pentanecarbonitrile | 167 | C₁₀H₁₇NO | ND | ND | ND | ND | ND | ND | 0.54 | 28.72 | ND | ND | ND | ND |
| (2"S,Sa,2"'S)-1,1-Dihydroxy-3,3-bis[2-hydroxy -1-propyl]-2,2',4,4',6,6'- hexamethoxy-5,5'-naphthalene | 582 | C₃₂H₃₈O₁₀ | ND | ND | ND | ND | ND | ND | ND | ND | ND | ND | 0.48 | 42.50 |
| (2-decyldodecyl), benzene | 386 | C_28_H_50_ | ND | ND | 0.62 | 24.52 | ND | ND | ND | ND | ND | ND | ND | ND |
| (9Z,12Z,15Z)-[9,10,12, 13,15,16,17,17-2H8]-1-(Tetrahydropyran-2'-yloxy)octadeca-9,12,15-triene | 348 | C_23_H_32_D_8_O_2_ | 0.71 | 49.93 | ND | ND | ND | ND | ND | ND | ND | ND | ND | ND |
| [3-(2-Cyclohexylethyl)-6-cyclopentylhexyl], benzene | 340 | C₂₅H₄₀ | ND | ND | 0.38 | 41.73 | ND | ND | 0.37 | 43.49 | ND | ND | ND | ND |
| 1-(1,5-Dimethylhexyl)-3A,6,12A-trimethyl tetradeca hydro-1H-cyclopenta[A]cyclopropa [E]phenanthren-7-YL acetate | 456 | C_31_H_51_DO_2_ | ND | ND | 0.21 | 51.66 | ND | ND | ND | ND | ND | ND | ND | ND |
| 1-(4-Nitrophenyl)-3,6-diazahomoadamantan-9 -one | 287 | C₁₅H₁₇N₃O₃ | ND | ND | ND | ND | ND | ND | ND | ND | ND | ND | 0.64 | 47.31 |
| 1,1'- [4-(3-Phenylpropyl)-1,7-heptanediyl]bis, benzene | 370 | C_28_H_34_ | 0.52 | 37.85 | ND | ND | ND | ND | ND | ND | ND | ND | ND | ND |
| 1,1,1-Trichloro-4 (dimethylamino) 3-[1-(4-fluorophenyl)-1H-tetraazol-5-YL]-3-buten-2 one | 377 | C_13_H_11_C_l3_FN_5_O | ND | ND | 0.31 | 48.92 | ND | ND | ND | ND | ND | ND | ND | ND |
| 1,1,6,6-Tetramethylspiro [4.4] nonane | 180 | C₁₃H₂₄ | ND | ND | ND | ND | ND | ND | 0.68 | 21.16 | ND | ND | ND | ND |
| 1,1'-dodecylidenebis- [4-methyl- Cyclohexane. | 362 | C₂₆H₅₀ | 2.95 | 40.88 | ND | ND | ND | ND | ND | ND | ND | ND | 0.58 | 26.37 |
| 1,2,5-Cyclodecatriene | 134 | C_10_H_14_ | ND | ND | 0.40 | 37.91 | ND | ND | ND | ND | ND | ND | ND | ND |
| 1,6-Dibromo-2-cyclohexylpentane | 310 | C_11_H_20_Br_2_ | ND | ND | ND | ND | 0.3 | 20.07 | ND | ND | ND | ND | ND | ND |
| 1,7 -Di one- Spiro[5.5]undecane | 180 | C₁₁H₁₆O₂ | ND | ND | ND | ND | ND | ND | ND | ND | 0.28 | 21.16 | ND | ND |
| 10-Methyl- eicosane | 296 | C₂₁H₄₄ | ND | ND | ND | ND | ND | ND | ND | ND | 0.78 | 25.90 | ND | ND |
| 11-Decyl- docosane | 450 | C₃₂H₆₆ | ND | ND | ND | ND | ND | ND | ND | ND | ND | ND | 3.60 | 37.75 |
| 3 Acetoxy-7,8-epoxylanostan,11-ol | 502 | C₃₂H₅₄O₄ | ND | ND | ND | ND | ND | ND | ND | ND | ND | ND | 0.63 | 54.02 |
| 13-Oxabicyclo [10.1.0]tridecane | 182 | C₁₂H₂₂O | ND | ND | ND | ND | ND | ND | ND | ND | 0.27 | 23.00 | ND | ND |
| 14-Methyl-, methyl ester, pentadecanoic acid | 270 | C_17_H_34_O_2_ | 0.46 | 36.47 | ND | ND | ND | ND | ND | ND | ND | ND | ND | ND |
| 16,16-D2-androst-5 | 276 | C_19_H_30_D_2_O | ND | ND | ND | ND | 0.41 | 21.99 | ND | ND | ND | ND | ND | ND |
| 17a-Ethyl-3á-methoxy- 17a-aza-D-homoandrost-5-ene-17-one | 345 | C₂₂H₃₅NO₂ | ND | ND | ND | ND | ND | ND | ND | ND | ND | ND | 0.93 | 30.75 |
| 17-Pentatriacontene | 490 | C_35_H_70_ | ND | ND | 0.21 | 44.56 | ND | ND | ND | ND | ND | ND | ND | ND |
| 1a,2,5,5a,6,9,10,10a-oc tahydro-5a-hydroxy-4-( hydroxymethyl)-1,1,7,9 tetramethyl-6,11-dioxo 1H-2,8a-methanocyclo penta[a]cyclopropa[e]cy clodecen-5-yl ester, [1aR-(1aà,2à,5á,5aá,8a à,9à,10aà)], dodecanoic acid | 528 | C_32_H_48_O_6_ | ND | ND | 0.21 | 47.23 | ND | ND | ND | ND | ND | ND | ND | ND |
| 1-Azabicyclo [2.2.2] octan-3-one oxime | 140 | C_7_H_12_N_2_O | ND | ND | ND | ND | 0.29 | 23.15 | ND | ND | ND | ND | ND | ND |
| 1-Cyclohexylheptene | 180 | C₁₃H₂₄ | ND | ND | ND | ND | ND | ND | 0.41 | 23.16 | ND | ND | ND | ND |
| 1-Decylbenzene | 218 | C_16_H_26_ | ND | ND | ND | ND | 0.44 | 31.33 | ND | ND | ND | ND | ND | ND |
| 5-Butyl-6-hexyloctahydro-1H-Indene | 264 | C₁₉H₃₆ | ND | ND | ND | ND | ND | ND | ND | ND | ND | ND | 0.67 | 28.74 |
| 1-Tert-butyl-3-(1-methyl cyclohexyl)-2-aziridinone | 209 | C_13_H_23_NO | ND | ND | ND | ND | 0.39 | 15.72 | ND | ND | ND | ND | ND | ND |
| 1-Tetradecanol | 214 | C₁₄H₃₀O | ND | ND | ND | ND | ND | ND | ND | ND | 1.05 | 30.23 | ND | ND |
| 2-(2,4-Dinitrophenylthio )-, methyl ester, benzoic acid | 334 | C₁₄H₁₀N₂O₆S | ND | ND | ND | ND | ND | ND | ND | ND | ND | ND | 0.50 | 27.18 |
| Trans-1H-Indene, octahydro-2,2,4,4,7,7-hexamethyl | 208 | C₁₅H₂₈ | ND | ND | ND | ND | ND | ND | ND | ND | 0.26 | 25.36 | ND | ND |
| 2,2,4,9,11,11-Hexamethyl, dodecane | 254 | C₁₈H₃₈ | ND | ND | ND | ND | ND | ND | 3.36 | 26.84 | 0.56 | 14.86 | ND | ND |
| 2,3,4,5-Tetrahydro-3-pyridazinone, 6-phenyl(deuterate) | 174 | C₁₀H₅D₅N₂O | ND | ND | ND | ND | ND | ND | 0.39 | 22 | ND | ND | ND | ND |
| 2,3-Dihydroxypropyl ester, hexadecanoic acid | 330 | C_19_H_38_O_4_ | 3.1 | 37.54 | ND | ND | ND | ND | ND | ND | ND | ND | ND | ND |
| 2,3-Dimethyl, nonadecane | 296 | C₂₁H₄₄ | ND | ND | ND | ND | ND | ND | ND | ND | 0.36 | 38.9 | ND | ND |
| 2,4,6(1H,3H ,5H)-Trione, 5-(1-phenylhydrazono) ethyl- pyrimidine | 260 | C₁₂H₁₂N₄O₃ | ND | ND | ND | ND | ND | ND | 1.22 | 37.86 | ND | ND | ND | ND |
| 2,4-Dimethyl, pentane | 100 | C₇H₁₆ | ND | ND | ND | ND | ND | ND | ND | ND | 1.09 | 21.63 | ND | ND |
| 2,4-Dimethyldodecane | 198 | C₁₄H₃₀ | ND | ND | ND | ND | ND | ND | ND | ND | 1.97 | 24.31 | ND | ND |
| 2,5-Dimethyl, undecane | 184 | C_13_H_28_ | ND | ND | ND | ND | 0.69 | 19.04 | ND | ND | ND | ND | ND | ND |
| 2,6,10,15,19,23-Hexamethyl,2,6,10,14,18,22-tetracosahexaene | 410 | C₃₀H₅₀ | ND | ND | ND | ND | ND | ND | ND | ND | ND | ND | 1.78 | 51.15 |
| 2,6,10-Trimethyl, dodecane | 212 | C₁₅H₃₂ | ND | ND | ND | ND | ND | ND | 1.50 | 23.56 | 0.75 | 23.56 | ND | ND |
| 2,6,10-Trimethyl, tetradecane | 240 | C₁₇H₃₆ | ND | ND | ND | ND | ND | ND | 1.17 | 20.71 | ND | ND | ND | ND |
| 2á,4a-Epoxymethylphenanthrene-7-methanol, 1,1-dimethyl-2-methoxy-8-(1,3-dithiin-2-ylidene) methyl-1,2,3,4,4a,4b,5,6,7,8,8a,9-dodecahydro-, acetate | 490 | C₂₇H₃₈O₄S₂ | ND | ND | ND | ND | ND | ND | ND | ND | ND | ND | 0.48 | 47.46 |
| 2-Butyl pentadecyl ester, fumaric acid | 382 | C_23_H_42_O_4_ | ND | ND | 0.40 | 45.75 | ND | ND | ND | ND | ND | ND | ND | ND |
| 2-Butyl-1,3,2-oxazaborolane | 127 | C_6_H_14_BNO | ND | ND | 4.60 | 31.53 | ND | ND | ND | ND | ND | ND | ND | ND |
| 2-Methoxy-2,3,6-trimethyl-1-oxo-2-silacyclohexa-3,5-diene | 170 | C_8_H_14_O_2_Si | ND | ND | ND | ND | 0.27 | 27.93 | ND | ND | ND | ND | ND | ND |
| 2-Methyl-, 2 (acetyloxy)-1,1a,2,3, 4,6,7,10,11,11a-26decahydro-7,10-dihydroxy-1,1, 3,9-pentamethyl-4a,7a -epoxy-5H-cyclopenta[a]cyclopropa[f]cycloundecen-11-yl ester,[1aR-[1aR*,2R*,3S*,4aR*,6S*,7S*,7aS*,8E,10R*,11R*(E),11aS*]]-2-butenoic acid | 490 | C₂₇H₃₈O₈ | ND | ND | ND | ND | ND | ND | ND | ND | 0.26 | 36.72 | ND | ND |
| 2-Methyl, decane | 156 | C_11_H_24_ | ND | ND | 0.86 | 26.93 | ND | ND | ND | ND | ND | ND | ND | ND |
| 2-Methyl, dodecane | 184 | C_13_H_28_ | ND | ND | ND | ND | 0.82 | 20.54 | ND | ND | ND | ND | ND | ND |
| 2-Methyl, heptadecane | 254 | C_18_H_38_ | ND | ND | ND | ND | 0.91 | 32.87 | ND | ND | ND | ND | ND | ND |
| 2-Methyl, hexadecane | 240 | C_17_H_36_ | ND | ND | ND | ND | 1.57 | 30.67 | ND | ND | ND | ND | ND | ND |
| 2-Methyl, octadecane | 268 | C_19_H_40_ | 0.28 | 33.05 | ND | ND | 0.77 | 34.97 | ND | ND | ND | ND | ND | ND |
| 2-Methyl, pentadecane | 226 | C_16_H_34_ | ND | ND | ND | ND | 2.10 | 28.34 | 1.60 | 28.35 | ND | ND | ND | ND |
| 2-Methyl-cis-7,8-epoxy nonadecane | 296 | C₂₀H₄₀O | ND | ND | ND | ND | ND | ND | ND | ND | ND | ND | 0.48 | 28.41 |
| 4-Cyclohexyliden-3,3-diethyl,2-pentanone | 222 | C_15_H_26_O | ND | ND | ND | ND | 0.29 | 24.75 | ND | ND | ND | ND | ND | ND |
| 3,5-Dideutero-aniline | 93 | C₆H₅D₂N | ND | ND | ND | ND | ND | ND | ND | ND | ND | ND | 0.60 | 37.23 |
| 3,7,11-Trimethyl-1-dodecanol | 228 | C_15_H_32_O | 0.40 | 35.44 | ND | ND | ND | ND | ND | ND | ND | ND | ND | ND |
| 3,9-dimethyl, undecane | 184 | C_13_H_28_ | ND | ND | ND | ND | 1.28 | 23.29 | ND | ND | ND | ND | ND | ND |
| 3-Dimethyl (trimethyl silylmethyl) silyloxypenta decane | 372 | C_21_H_48_OSi_2_ | 0.48 | 34.51 | ND | ND | ND | ND | ND | ND | ND | ND | ND | ND |
| 3-Ethoxycarbonyl-1-hydroxy-4-azaindol-2(3H)-one | 222 | C₁₀H₁₀N₂O₄ | ND | ND | ND | ND | ND | ND | ND | ND | 0.30 | 28.73 | ND | ND |
| 3-Ethyl-5-(2-ethylbutyl), octadecane | 366 | C_26_H_54_ | 0.33 | 42.45 | 0.31 | 48.81 | ND | ND | 1.06 | 28.02 | ND | ND | 2.24 | 29.34 |
| 4,25-Secoobscurinervan ,21-deoxy-16-methoxy-2 2-methyl-, (22à)- (CAS) | 368 | C₂₃H₃₂N₂O₂ | ND | ND | 0.38 | 42.48 | ND | ND | ND | ND | ND | ND | 0.93 | 35.02 |
| 4-Methyl, hexadecane | 240 | C₁₇H₃₆ | ND | ND | ND | ND | 2.24 | 25.89 | ND | ND | 1.43 | 30.68 | ND | ND |
| 4-Methyl, pentadecane | 226 | C_16_H_34_ | ND | ND | ND | ND | 0.29 | 26.07 | ND | ND | ND | ND | ND | ND |
| 4-Methyl, tridecane | 198 | C_14_H_30_ | 6.92 | 33.69 | ND | ND | ND | ND | ND | ND | ND | ND | ND | ND |
| 5,14-Dibutyl, octadecane | 366 | C₂₆H₅₄ | ND | ND | ND | ND | ND | ND | 0.38 | 40.72 | ND | ND | ND | ND |
| 5,15-Dimethyl, nonadecane | 296 | C₂₁H₄₄ | ND | ND | ND | ND | ND | ND | ND | ND | ND | ND | 5.26 | 46.37 |
| 5,6,7,8-Tetramethyl-1,2, 3,4-tetraphenyl-, cyclooctatetraene | 464 | C₃₆H₃₂ | ND | ND | ND | ND | ND | ND | 0.45 | 53.95 | ND | ND | ND | ND |
| 5,8-Diethyl, dodecane | 226 | C_16_H_34_ | 0.47 | 25.90 | ND | ND | ND | ND | ND | ND | ND | ND | ND | ND |
| 6-Ethenyl-2,4,5,6,7,7a hexahydro-3,6-dimethyl-à-methylene-2-oxo-, methyl ester,5-benzofuranacetic acid | 276 | C₁₆H₂₀O₄ | ND | ND | ND | ND | ND | ND | ND | ND | 0.30 | 31.36 | ND | ND |
| 5-Butyl, hexadecane | 282 | C₂₀H₄₂ | ND | ND | ND | ND | ND | ND | ND | ND | 1.18 | 34.99 | ND | ND |
| 5-Methyl, tetradecane | 212 | C_15_H_32_ | ND | ND | ND | ND | 1.98 | 17.62 | ND | ND | 6.33 | 23.31 | ND | ND |
| 5-Tert-butyl-2,3-dimethoxytoluene | 208 | C₁₃H₂₀O₂ | ND | ND | ND | ND | ND | ND | 0.54 | 25.36 | ND | ND | ND | ND |
| 6,9-Dimethyl, tetradecane | 226 | C₁₆H₃₄ | ND | ND | ND | ND | ND | ND | 0.45 | 23.31 | ND | ND | ND | ND |
| 6-[(2'-Furyl) methylene] -tetrahydropyran-2-one | 178 | C₁₀H₁₀O₃ | ND | ND | ND | ND | ND | ND | 0.48 | 21.40 | ND | ND | ND | ND |
| 6-Tetradecanesulfonic acid, butyl ester | 334 | C₁₈H₃₈O₃S | ND | ND | ND | ND | ND | ND | 1.5 | 30.68 | ND | ND | ND | ND |
| 7-(1-Bromoethyl)-3,3-dimethyl-bicyclo [4.1.0] heptane-2-one | 244 | C_11_H_17_BrO | ND | ND | ND | ND | 0.45 | 21.38 | ND | ND | ND | ND | ND | ND |
| 7,10-Pentadecadiynoic acid | 234 | C_15_H_22_O_­_ | ND | ND | 0.77 | 39.86 | ND | ND | ND | ND | ND | ND | ND | ND |
| 7,3',4'-Trimethoxy quercetin | 344 | C₁₈H₁₆O₇ | ND | ND | ND | ND | ND | ND | ND | ND | ND | ND | 1.14 | 45.68 |
| 7,8-Dihydro-6H-benzo [3,4] cyclobuta [1,2]cycloheptene(5,9-D2) | 168 | C₁₃H₁₀D₂ | ND | ND | ND | ND | ND | ND | 0.45 | 30.01 | ND | ND | ND | ND |
| 7aHCyclopenta[a]cyclopropa[f]cycloundecene -2,4,7,7a,10,11-hexol, 1,1a,2,3,4,4a,5,6,7,10,1 1,11a-dodecahydro-1,1, 3,6,9 pentamethyl-,2,4,7,10,11-pentaacetate | 580 | C₃₀H₄₄O₁₁ | ND | ND | ND | ND | ND | ND | ND | ND | ND | ND | 0.83 | 53.30 |
| 7-Methyl, pentadecane | 226 | C₁₆H₃₄ | ND | ND | ND | ND | ND | ND | ND | ND | 1.15 | 25.75 | ND | ND |
| 7-Methyl, tridecane | 198 | C_14_H_30_ | ND | ND | ND | ND | 1.76 | 20.71 | ND | ND | ND | ND | ND | ND |
| 7-Methyl, octadecane | 268 | C_19_H_40_ | ND | ND | ND | ND | 1.13 | 30.22 | ND | ND | 1.37 | 28.35 | ND | ND |
| 8-(N-Ethanoylamido)-5,6-dimethoxyquinoline | 260 | C_14_H_16_N_2_O_3_ | 0.36 | 34.34 | ND | ND | ND | ND | ND | ND | ND | ND | ND | ND |
| 9-Desoxo-9-x-acetoxy-3,8,12-tri-O-acetylingol | 536 | C₂₈H₄₀O₁₀ | ND | ND | ND | ND | ND | ND | ND | ND | ND | ND | 0.61 | 40.51 |
| 9-Hexyl, heptadecane | 324 | C_23_H_48_ | 0.45 | 36.98 | 0.31 | 37.00 | ND | ND | 1.53 | 32.88 | 1.29 | 32.89 | ND | ND |
| 9-Methyl, acridine | 193 | C_14_H_11_N | ND | ND | ND | ND | 0.71 | 26.36 | ND | ND | ND | ND | ND | ND |
| 9-Octyl, heptadecane | 352 | C₂₅H₅₂ | ND | ND | ND | ND | ND | ND | ND | ND | 0.45 | 36.99 | ND | ND |
| 13-Acetoxymethyl-17-acetyl-9-hydroxy-10-methyl-3-oxo-2,3,6,7,8,9,10,11,12,13,14,15,16,17- tetradecahydro-1H-cyclopenta[a]phenanthrene, acetic acid | 446 | C₂₅H₃₄O₇ | ND | ND | ND | ND | ND | ND | ND | ND | ND | ND | 1.34 | 53.73 |
| 9a-[(Acetyloxy)methyl] decahydro6a,9-dihydroxy-6methyl-3-methylene-, [3aS(3aà,6á,6aà,9á,9aá,9bà)]-azuleno[4,5-b]furan-2(3H)-one | 324 | C₁₇H₂₄O₆ | ND | ND | ND | ND | ND | ND | ND | ND | ND | ND | 0.77 | 46.9 |
| Cadmium chloride porphine derivative complex | 607 | C₂₉H₄₀CdClN₅ | ND | ND | 0.21 | 52.77 | ND | ND | ND | ND | ND | ND | 1.54 | 49.72 |
| Cantaxanthin | 564 | C_40_H_52_O_2_ | 0.26 | 48.95 | ND | ND | ND | ND | ND | ND | ND | ND | ND | ND |
| Ceanothine C | 470 | C₂₆H₃₈N₄O₄ | ND | ND | ND | ND | ND | ND | ND | ND | ND | ND | 0.63 | 51.63 |
| Diethyl-, 3,3,5-trimethyl-1-cycloh exen-1-yl ester, Borinic acid | 208 | C_13_H_25_BO | ND | ND | ND | ND | 0.30 | 25.36 | ND | ND | ND | ND | ND | ND |
| Dihydroxanthin | 308 | C₁₇H₂₄O₅ | ND | ND | ND | ND | ND | ND | ND | ND | ND | ND | 0.74 | 40.65 |
| Docosane | 310 | C₂₂H₄₆ | 2.04 | 30.67 | 18.49 | 25.78 | 3.82 | 28.01 | 27.44 | 19.06 | 10.65 | 26.84 | 29.36 | 23.62 |
| Dodecane | 170 | C₁₂H₂₆ | ND | ND | ND | ND | 1.42 | 18.72 | ND | ND | 0.23 | 19.05 | ND | ND |
| Dotriacontane | 450 | C_32_H_66_ | ND | ND | 0.51 | 44.15 | ND | ND | ND | ND | ND | ND | ND | ND |
| Eicosane | 282 | C₂₀H₄₂ | 4.64 | 37.72 | ND | ND | 2.89 | 37.73 | 5.11 | 37.73 | 5.47 | 37.73 | ND | ND |
| Ethyl phenethyl ester, diethylmalonic acid | 292 | C_17_H_24_O_4_ | 0.35 | 36.20 | ND | ND | ND | ND | ND | ND | ND | ND | ND | ND |
| Heneicosane | 296 | C₂₁H₄₄ | 5.55 | 39.59 | ND | ND | 3.11 | 39.61 | 4.88 | 39.61 | ND | ND | ND | ND |
| Heptacosane | 380 | C₂₇H₅₆ | ND | ND | ND | ND | 3.72 | 49.40 | 4.17 | 44.78 | 4.04 | 49.38 | ND | ND |
| Heptadecane | 240 | C_17_H_36_ | 7.58 | 31.51 | ND | ND | 7.99 | 31.55 | ND | ND | 8.55 | 31.53 | ND | ND |
| Hexacosane | 366 | C_26_H_54_ | ND | ND | 7.37 | 47.90 | 3.47 | 47.92 | ND | ND | 4.11 | 47.91 | ND | ND |
| Hexadecane | 226 | C_16_H_34_ | 3.73 | 29.24 | ND | ND | 6.15 | 29.26 | 4.37 | 29.25 | 4.67 | 29.25 | ND | ND |
| Hexadecyl, benzene | 302 | C₂₂H₃₈ | ND | ND | ND | ND | ND | ND | ND | ND | 0.42 | 43.51 | ND | ND |
| Hexatriacontane | 506 | C₃₆H₇₄ | 16.84 | 35.75 | ND | ND | 3.89 | 46.39 | 4.20 | 43.13 | ND | ND | 7.18 | 47.9 |
| L-Lysine, N6-acetyl-N2-[N-[N-[ N-(N2-acetyl-N,N,N2-trimethyl-L-asparaginyl) N-methyl-L-phenylalanyl]-N-methyl-L-phenylalanyl]-N,1-dimethyl-L-tryptophyl]-N2,N6-dimethyl-, methyl ester | 964 | C_53_H_72_N_8_O_9_ | ND | ND | 0.31 | 52.86 | ND | ND | ND | ND | ND | ND | ND | ND |
| Lucenin 2 | 610 | C₂₇H₃₀O₁₆ | ND | ND | ND | ND | ND | ND | ND | ND | ND | ND | 0.96 | 47.23 |
| Methyl 12-isopropenylidene-6, 8a-cyclopenteno-2H-di hydrocyclohepta[b]furan-2-one-3-carboxylate | 310 | C_19_H_18_O_4_ | 0.35 | 28.96 | ND | ND | ND | ND | ND | ND | ND | ND | ND | ND |
| 13-Chloro-5-demethoxy-28-deoxy-6,28-epoxy- 5-(hydroxyimino)-25-(1-methylethyl)-, (6R,13R,25R)-milbemycin b | 603 | C₃₃H₄₆ClNO_₇_ | ND | ND | ND | ND | ND | ND | ND | ND | ND | ND | 0.74 | 49.81 |
| Minovincinine | 354 | C₂₁H₂₆N₂O₃ | ND | ND | ND | ND | ND | ND | ND | ND | ND | ND | 0.51 | 40.87 |
| N-(2-Pyridinyl)-2-methyl aniline | 184 | C_12_H_12_N_2_ | 0.80 | 30.22 | ND | ND | ND | ND | ND | ND | ND | ND | ND | ND |
| N'-(2-Pyrrolidino-5-nitrobenzylidene)-2-(para-tolyloxy) acethydrazide | 382 | C₂₀H₂₂N₄O₄ | ND | ND | ND | ND | ND | ND | ND | ND | 0.59 | 28.97 | ND | ND |
| Nonacosane | 408 | C_29_H_60_ | 3.92 | 38.88 | 7.08 | 47.30 | 2.03 | 52.56 | 2.12 | 52.53 | 0.33 | 42.48 | 10.99 | 25.79 |
| Nonadecane | 268 | C₁₉H₄₀ | ND | ND | 4.29 | 35.76 | 7.07 | 25.75 | ND | ND | 7.02 | 35.77 | ND | ND |
| Nonyl- benzene | 204 | C₁₅H₂₄ | ND | ND | ND | ND | ND | ND | 1.2 | 28.96 | ND | ND | ND | ND |
| Octacosane | 394 | C₂₈H₅₈ | 3.97 | 50.84 | 6.55 | 50.84 | 2.68 | 50.86 | 2.35 | 50.84 | 2.95 | 50.85 | ND | ND |
| Octadecane | 254 | C₁₈H₃₈ | 0.63 | 25.75 | 4.44 | 33.71 | 5.25 | 33.70 | 6.74 | 33.7 | 7.3 | 33.7 | ND | ND |
| Octahydro-1-(2-octyldecyl)-pentalene | 362 | C_26_H_50_ | ND | ND | ND | ND | 0.7 | 22.98 | ND | ND | ND | ND | ND | ND |
| Pentacosane | 352 | C₂₅H₅₂ | 6.25 | 28.35 | ND | ND | ND | ND | 5.53 | 30.23 | 5.01 | 46.38 | ND | ND |
| Pentadecane | 212 | C_15_H_32_ | 2.2 | 26.83 | ND | ND | 6.38 | 26.85 | ND | ND | ND | ND | ND | ND |
| Pentadecyl- benzene | 288 | C₂₁H₃₆ | ND | ND | ND | ND | ND | ND | ND | ND | 0.4 | 41.72 | ND | ND |
| Pentatriacontane | 492 | C_35_H_72_ | ND | ND | 5.11 | 39.61 | ND | ND | ND | ND | ND | ND | ND | ND |
| Serotonin | 176 | C₁₀H₁₂N₂O | ND | ND | ND | ND | ND | ND | ND | ND | ND | ND | 0.62 | 26.95 |
| Tert-Hexadecanethiol | 258 | C₁₆H₃₄S | ND | ND | ND | ND | ND | ND | ND | ND | ND | ND | 0.55 | 32.92 |
| Tetracosane | 338 | C₂₄H₅₀ | 5.72 | 44.78 | 7.20 | 44.78 | 3.80 | 44.79 | ND | ND | 11.06 | 43.14 | 5.37 | 44.78 |
| Tetradecane | 198 | C₁₄H₃₀ | 0.99 | 24.30 | ND | ND | 5.91 | 24.30 | 2.26 | 24.30 | ND | ND | ND | ND |
| Tetradecyl- benzene | 274 | C₂₀H₃₄ | ND | ND | ND | ND | 0.48 | 39.81 | 0.94 | 39.83 | 0.93 | 39.84 | ND | ND |
| Tetratetracontane | 618 | C_44_H_90_ | 0.29 | 48.79 | 8.59 | 49.38 | ND | ND | 1.22 | 54.55 | ND | ND | 9.28 | 49.37 |
| Tetratriacontane | 478 | C_34_H_70_ | 3.34 | 52.54 | 3.13 | 54.56 | ND | ND | 6.45 | 47.9 | ND | ND | ND | ND |
| Trans-2H-Benzocyclohepten-2-one, decahydro-9a-methyl-, | 180 | C₁₂H₂₀O | ND | ND | ND | ND | 0.66 | 21.15 | 0.39 | 23.00 | ND | ND | ND | ND |
| Triacontane | 422 | C₃₀H₆₂ | 9.96 | 47.90 | ND | ND | 1.14 | 54.59 | ND | ND | 1.37 | 54.57 | ND | ND |
| Tricosane | 324 | C_23_H_48_ | ND | ND | 14.36 | 43.13 | 3.38 | 43.13 | 2.53 | 25.75 | ND | ND | 4.54 | 43.13 |
| Tridecane | 184 | C_13_H_28_ | 0.27 | 23.56 | ND | ND | 3.56 | 21.59 | ND | ND | ND | ND | ND | ND |
| Tridecyl- benzene | 260 | C₁₉H₃₂ | ND | ND | ND | ND | 0.30 | 37.85 | ND | ND | 0.69 | 37.87 | ND | ND |
| Trihexadecylborate | 734 | C_48_H_99_BO_3_ | 1.00 | 34.98 | ND | ND | ND | ND | ND | ND | ND | ND | ND | ND |
| Tritetracontane | 604 | C_43_H_88_ | ND | ND | 1.17 | 53.95 | ND | ND | ND | ND | ND | ND | ND | ND |
| Zearalenone | 318 | C₁₈H₂₂O₅ | ND | ND | ND | ND | ND | ND | ND | ND | ND | ND | 1.16 | 27.00 |

R_t_, retention time; MF, molecular formula; MW, molecular weight; ND, not detected.

**Table S11** Cultural characteristics of the 28 actinobacterial isolates on starch nitrate agar, ISP2, ISP3 and ISP4 media.

| Isolate | Appearance | Starch nitrate agar | | | ISP2 | | | ISP3 | | | ISP4 | | |
| --- | --- | --- | --- | --- | --- | --- | --- | --- | --- | --- | --- | --- | --- |
|  |  | Aerial mycelium | Substrate mycelium | Diffusible pigment | Aerial mycelium | Substrate mycelium | Diffusible pigment | Aerial mycelium | Substrate mycelium | Diffusible pigment | Aerial mycelium | Substrate mycelium | Diffusible pigment |
| A1 | V | Grey with white edge | Brown | - | Grey | Beige | - | Grey | Brown | - | Grey | Black | - |
| A2 | V | Grey with white edge | Black | Brown | Grey | Creamy white | - | Pale grey | Brown | - | Grey | Black | - |
| A3 | V | Grey | Black | - | Grey | Creamy white | - | Pale grey | Brown | - | Grey | Creamy white | - |
| A4 | V | Grey | Black | Brown | Grey | Brown | - | Grey | Yellow | - | Grey | Beige | - |
| A5 | V | Grey | Black | - | Grey | Brown | - | No aerial growth | Brown | - | Grey | Brown | - |
| A6 | V | Grey with white edge | Beige | - | Grey | Beige | - | Grey | Beige | - | Grey | Beige | - |
| A7 | V | Grey with white edge | Salmon red | Brown | White | Beige | - | Grey | Beige | - | Grey | Brown | - |
| A8 | V | Grey | Salmon red | Brown | Grey | Beige | - | Grey | Beige | - | Grey | Brown | - |
| A9 | P | Grey | Black | - | Grey | Beige | - | Grey | Black | Brown | Grey | Brown | - |
| A10 | V | Grey | Beige | - | Grey | Beige | - | Grey | Black | Brown | Grey | Beige | - |
| A11 | V | Grey | Beige | - | Grey | Beige | - | Grey | Black | Brown | Grey | Beige | - |
| A12 | V | Grey | Brown | - | Grey | Black | - | Grey | Black | - | Grey | Beige | - |
| A13 | P | Grey with white edge | Black | - | Grey | Beige | - | No aerial growth | Brown | - | Grey | Creamy white | - |
| A14 | P | Grey | Beige | - | Grey | Beige | - | Grey | Black | Brown | Grey | Beige | - |
| A15 | P | Grey | Beige | - | Grey | Beige | - | Grey | Brown | Brown | Grey | Beige | - |
| B1 | V | Grey | Creamy white | - | Grey | Beige | - | Grey | Creamy white | - | Grey | Creamy white | - |
| B2 | P | Grey | Beige | - | Grey | Beige | - | Grey | Creamy white | - | Grey | Creamy white | - |
| B3 | C | Grey | Yellow | - | Grey | Beige | - | White | Beige | - | White | Creamy white | - |
| B4 | P | Grey | Beige | - | Grey | Creamy white | - | Grey | Creamy white | - | No aerial growth | Creamy white | - |
| B5 | C | Grey | Beige | - | White | Yellow | - | Grey | Beige | - | Grey | Creamy white | - |
| B6 | P | Grey | Beige | - | Grey | Dark Brown | - | Grey | Dark brown | - | Grey | Beige | - |
| C1 | V | Grey | Beige | - | Pale grey | Beige | - | Grey | Creamy white | - | Grey | Creamy white | - |
| C2 | V | Grey with white edge | Salmon red | - | Grey | Beige | - | Grey | Beige | - | Grey | Beige | - |
| C3 | V | Grey | Beige | - | Grey | Beige | - | Grey | Brown | - | Grey | Creamy white | - |
| C4 | V | Grey | Beige | - | Grey | Yellow | - | Grey | Black | - | Grey | Beige | - |
| C5 | V | Grey | Beige | - | Grey | Yellow | - | Grey | Black | - | Pale grey | Beige | - |
| C6 | V | Grey | Yellow | - | Grey | Yellow | - | Grey | Beige | - | Grey | Creamy white | - |
| C7 | C | Grey | Beige | - | Grey | Yellow | - | Grey | Beige | - | Grey | Beige | - |

C, Cottony; V, velvety; P, powdery; +, growth or pigment produced; -, no pigment produced.

**Table S12** Cultural characteristics of the 28 actinobacterial isolates on ISP5, ISP6 and ISP7 media.

| Isolate | ISP5 | | | ISP6 | | | ISP7 | | |
| --- | --- | --- | --- | --- | --- | --- | --- | --- | --- |
|  | Aerial mycelium | Substrate mycelium | Diffusible pigment | Aerial mycelium | Substrate mycelium | Diffusible pigment | Aerial mycelium | Substrate mycelium | Diffusible pigment |
| A1 | No aerial growth | Black | - | Grey | Creamy white | - | No aerial growth | Brown | - |
| A2 | Grey | Black | - | Grey | Beige | - | No aerial growth | Beige | - |
| A3 | Grey | Brown | - | Grey | Creamy white | - | Grey | Creamy white | - |
| A4 | Pale Grey | Brown | - | Grey | Beige | - | Grey | Creamy white | - |
| A5 | Grey | Brown | - | Grey | Beige | - | No aerial growth | Buff | - |
| A6 | Grey | Beige | - | Grey | Beige | - | Grey | Creamy white | - |
| A7 | Grey | Brown | - | Grey | Beige | - | Grey | Creamy white | - |
| A8 | Pale Grey | Brown | - | Grey | Beige | - | Grey | Creamy white | - |
| A9 | Grey | Brown | - | Grey | Creamy white | - | Grey | Creamy white | - |
| A10 | Grey | Beige | - | Grey | Beige | - | Grey | Beige | - |
| A11 | Grey | Brown | - | Grey | Beige | - | Grey | Brown | - |
| A12 | Grey | Brown | - | No aerial growth | Beige | - | No aerial growth | Yellow | - |
| A13 | Grey | Brown | - | No aerial growth | Beige | - | No aerial growth | Yellow | - |
| A14 | Grey | Brown | - | Grey | Brown | - | Grey | Brown | - |
| A15 | Grey | Dark Brown | Brown | Grey | Beige | - | Grey | Beige | - |
| B1 | No aerial growth | Brown | - | Grey | Beige | - | No aerial growth | Creamy white | - |
| B2 | Pale Grey | Beige | Brown | Grey | Beige | - | Pale grey | Brown | - |
| B3 | Pale Grey | Brown | - | Grey | Beige | - | Pale grey | Brown | - |
| B4 | No aerial growth | Beige | - | No aerial growth | Red | - | No aerial growth | Brown | - |
| B5 | Pale Grey | Beige | - | Grey | Beige | - | Grey | Beige | - |
| B6 | Grey | Beige | - | Grey | Beige | - | Grey | Beige | - |
| C1 | Pale Grey | Beige | - | White | Creamy white | - | No aerial growth | Creamy white | - |
| C2 | Grey + white | Beige | - | Grey | Beige | - | No aerial growth | Creamy white | - |
| C3 | No aerial growth | Beige | - | Grey | Beige | - | Grey | Beige | - |
| C4 | Grey | Beige | - | Grey | Beige | - | Grey | Beige | - |
| C5 | Pale grey | Beige | - | White | Beige | - | White | Creamy white | - |
| C6 | Pale grey | Brown | - | Pale grey | Beige | - | Pale grey | Beige | - |
| C7 | Pale grey | Beige | - | White | Beige | - | Pale grey | Beige | - |

-, no pigment produced.

**Table S13** Spore chains description of the tested actinobacteria

| Isolate | Spore chain length | Spore chain morphology | Spore shape | Spore surface |
| --- | --- | --- | --- | --- |
| A1 | Long | Mono-VE-S | Rods | Spiny |
| A2 | Short | Mono-VE-S | Rods | ND |
| A3 | Short | Mono-VE-RF | Rods | ND |
| A4 | Short | Mono-VE-RF | Rods | ND |
| A5 | Long | Mono-VE-RF | Spherical | ND |
| A6 | Long | Mono-VE-RF | Rod | ND |
| A7 | Long | Mono-VE-S | Rod | Smooth |
| A8 | Long | Mono-VE-S | Rod | ND |
| A9 | Long | Mono-VE-S | Rod | ND |
| A10 | Short | Mono-VE-S | Rod | ND |
| A11 | Short | Bi-VE-RF | Rod | ND |
| A12 | Long | Mono-VE-S | Rod | Spiny |
| A13 | Short | Bi-VE-S | Rod | ND |
| A14 | Short | Mono-VE-RF | Rod | ND |
| A15 | Short | Mono-VE-RF | Spherical | ND |
| B1 | Short | Mono-VE-RF | Rod | ND |
| B2 | Short | Bi-VE-S | Spherical | ND |
| B3 | Short | Mono-VE-RF | Rod | ND |
| B4 | Short | Mono-VE-S | Rod | ND |
| B5 | Short | Mono-VE-RF | Rod | ND |
| B6 | Short | Mono-VE-S | Rod | ND |
| C1 | Long | Mono-VE-RF | Rod | ND |
| C2 | Long | Mono-VE-S | Rod | ND |
| C3 | Short | Mono-VE-S | Rod | ND |
| C4 | Long | Mono-VE-RF | Rod | ND |
| C5 | Short | Mono-VE-S | Rod | ND |
| C6 | Short | Mono-VE-RF | Spherical | ND |
| C7 | Short | Mono-VE-RF | Spherical | ND |

Spore chain morphology indicated as RF, rectus flexibilis; S, spiral; VE, verticil; ND, not detected.

**Table S14** Utilization of different C sources by the tested actinobacteria

| Isolate | Carbon source | | | | | | | | | | | | |
| --- | --- | --- | --- | --- | --- | --- | --- | --- | --- | --- | --- | --- | --- |
|  | No carbon *(Negative control)* | D-Glucose  *(positive control)* | D-Fructose | Lactose | Galactose | Maltose | Mannose | Arabinose | D-Xylose | D-Mannitol | Inositol | Sucrose | Starch |
| A1 | - | + | + | - | - | + | + | + | + | + | + | + | + |
| A2 | - | + | + | - | - | + | + | + | + | + | + | + | + |
| A3 | - | + | + | - | - | + | + | + | + | + | + | + | + |
| A4 | - | + | + | - | + | + | + | + | + | + | + | + | + |
| A5 | - | + | + | + | + | + | + | + | + | + | + | + | + |
| A6 | - | + | + | + | + | + | + | + | + | + | + | + | + |
| A7 | - | + | + | + | + | + | + | + | + | + | + | + | + |
| A8 | - | + | + | + | + | + | + | + | + | + | + | + | + |
| A9 | - | + | + | + | + | + | + | + | + | + | + | + | + |
| A10 | - | + | + | + | + | + | + | + | + | + | + | + | + |
| A11 | - | + | + | + | + | + | + | + | + | + | + | + | + |
| A12 | - | + | + | + | + | + | + | + | + | + | + | + | + |
| A13 | - | + | + | + | + | + | + | + | + | + | + | + | + |
| B1 | - | + | + | + | + | + | + | + | + | + | + | + | + |
| B2 | - | + | W | w | + | + | + | - | w | W | - | + | + |
| B3 | - | + | + | + | + | + | + | + | + | + | + | + | + |
| B4 | - | + | W | w | + | + | + | - | w | W | + | + | + |
| B5 | - | + | + | + | + | + | + | + | + | + | + | + | + |
| B6 | - | + | + | + | + | + | + | + | + | + | + | + | + |
| C1 | - | + | + | + | + | + | + | + | + | + | + | + | + |
| C2 | - | + | + | + | + | + | + | + | + | + | + | + | + |
| C3 | - | + | + | + | + | + | + | + | + | + | + | + | + |
| C4 | - | + | + | + | + | + | + | + | + | + | + | + | + |
| C5 | - | + | + | + | + | + | + | + | + | + | + | + | + |
| C6 | - | + | + | + | + | + | + | + | + | + | + | + | + |
| C7 | - | + | + | + | + | + | + | + | + | + | + | + | + |

+, Growth; -, no growth; w, weak growth.

**Table S15** Biochemical characteristics and antagonistic properties of the tested actinobacteria

| Isolate | Sodium chloride tolerance (%) | | | | Growth at different pH degrees | | | Growth on Czapek’s agar | Sensitivity to streptomycin (50 µg mL^-1^) | Antimicrobial activity | | | | |
| --- | --- | --- | --- | --- | --- | --- | --- | --- | --- | --- | --- | --- | --- | --- |
|  | 2.5 | 5 | 7.5 | 10 | 5 | 7 | 9 |  |  | *Bacillus subtilis* | *Klebsiella* sp. | *Candida albicans* | *Aspergillus flavus* | *Penicillium* sp. |
| A1 | + | w | - | - | + | + | + | + | S | + | - | + | - | - |
| A2 | + | w | - | - | + | + | + | + | S | + | - | + | - | - |
| A3 | + | w | - | - | + | + | + | + | S | + | - | + | - | - |
| A4 | + | w | - | - | + | + | + | + | S | + | + | + | - | - |
| A5 | + | w | - | - | + | + | + | + | S | + | + | + | - | - |
| A6 | + | w | - | - | + | + | + | + | S | + | - | + | - | - |
| A7 | + | w | - | - | + | + | + | + | S | + | - | + | - | - |
| A8 | + | w | - | - | + | + | + | + | S | + | - | + | - | - |
| A9 | + | w | - | - | + | + | + | + | S | + | + | + | - | - |
| A10 | + | - | - | - | + | + | + | + | S | + | - | + | - | - |
| A11 | w | - | - | - | + | + | + | + | S | + | - | + | - | - |
| A12 | + | + | - | - | + | + | + | + | S | + | - | + | - | - |
| A13 | + | w | - | - | + | + | + | + | S | + | + | + | - | - |
| A14 | + | - | - | - | + | + | + | + | S | + | - | + | - | - |
| A15 | + | - | - | - | + | + | + | + | S | + | - | + | - | - |
| B1 | w | - | - | - | + | + | + | + | S | - | + | - | - | - |
| B2 | w | - | - | - | + | + | + | + | S | - | + | - | - | - |
| B3 | + | - | - | - | + | + | + | + | S | - | - | - | - | - |
| B4 | w | - | - | - | + | + | + | + | S | + | - | + | - | - |
| B5 | + | - | - | - | + | + | + | + | S | - | - | - | - | - |
| B6 | + | - | - | - | + | + | + | + | S | + | - | + | - | - |
| C1 | + | + | - | - | + | + | + | + | S | + | - | + | - | - |
| C2 | + | + | - | - | + | + | + | + | S | - | + | - | - | - |
| C3 | + | + | - | - | + | + | + | + | S | + | - | + | - | - |
| C4 | + | w | - | - | + | + | + | + | S | + | + | + | - | - |
| C5 | + | w | - | - | + | + | + | + | S | + | - | + | - | - |
| C6 | + | w | - | - | + | + | + | + | S | + | - | + | - | - |
| C7 | + | w | - | - | + | + | + | + | S | + | - | + | - | - |

+, Growth or inhibition zone; -, no growth or no inhibition zone; w, weak growth; S, sensitive.

**Table S16** Bioinformatics analysis as the DNA sequence of *Streptomyces* *aurantiogriseus* strain NORA7 was compared with sequences deposited in GenBank database using BLASTn algorithm

| Accession No. | Strain | Closest match (phenotypically) | Closest match ( genotypically) | | | | |
| --- | --- | --- | --- | --- | --- | --- | --- |
|  |  |  | Strain | Accession No. | Identity (%) | Query coverage (%) | E value |
| [PP851973](https://www.ncbi.nlm.nih.gov/nuccore/PP851973) | *Streptomyces* *aurantiogriseus* strain NORA7 | *Streptomyces* *aurantiogriseus* | [*Streptomyces viridosporus*](https://www.ncbi.nlm.nih.gov/Taxonomy/Browser/wwwtax.cgi?id=67581) CSSP718 | [NR_043366.1](https://www.ncbi.nlm.nih.gov/nucleotide/NR_043366.1?report=genbank&log$=nucltop&blast_rank=1&RID=4BZN17G2013) | 99.49 | 99 | 0 |
|  |  |  | [*Streptomyces geysiriensis*](https://www.ncbi.nlm.nih.gov/Taxonomy/Browser/wwwtax.cgi?id=68207) NBRC 15413 | [NR_112459.1](https://www.ncbi.nlm.nih.gov/nucleotide/NR_112459.1?report=genbank&log$=nucltop&blast_rank=2&RID=4BZN17G2013) | 99.49 | 99 | 0 |
|  |  |  | [*Streptomyces geysiriensis*](https://www.ncbi.nlm.nih.gov/Taxonomy/Browser/wwwtax.cgi?id=68207) NRRL B-12102 | [NR_043818.1](https://www.ncbi.nlm.nih.gov/nucleotide/NR_043818.1?report=genbank&log$=nucltop&blast_rank=1&RID=YAD2X0MC013) | 99.49 | 99 | 0 |
|  |  |  | [*Streptomyces minutiscleroticus*](https://www.ncbi.nlm.nih.gov/Taxonomy/Browser/wwwtax.cgi?id=68238) NRRL B-12202 | [NR_044149.1](https://www.ncbi.nlm.nih.gov/nucleotide/NR_044149.1?report=genbank&log$=nucltop&blast_rank=3&RID=4BZN17G2013) | 99.49 | 99 | 0 |
|  |  |  | *Streptomyces eneissocaesilis* NRRL B-16365 | [NR_115668.1](https://www.ncbi.nlm.nih.gov/nucleotide/NR_115668.1?report=genbank&log$=nucltop&blast_rank=4&RID=YAD2X0MC013) | 99.41 | 99 | 0 |
|  |  |  | *Streptomyces aurantiogriseus* NBRC 12842 | [NR_041081.1](https://www.ncbi.nlm.nih.gov/nucleotide/NR_041081.1?report=genbank&log$=nucltop&blast_rank=36&RID=4BZN17G2013) | 99.16 | 99 | 0 |
|  |  |  | *Streptomyces aurantiogriseus* CSSP546 | [NR_115394.1](https://www.ncbi.nlm.nih.gov/nucleotide/NR_115394.1?report=genbank&log$=nucltop&blast_rank=46&RID=4BZN17G2013) | 99.07 | 99 | 0 |
