# Supplementary material for: Statistical optimization of crude oil bioremediation using Streptomyces aurantiogriseus isolated from Egypt’s Western Desert
Source: Biodegradation. 2025 Jul 25;36(4):68. doi: 10.1007/s10532-025-10154-0 (PMC12296995; doi:10.1007/s10532-025-10154-0)
Supplement: Supplementary file 2 — Supplementary file2 (DOCX 1042 KB) [file 10532_2025_10154_MOESM2_ESM.docx]

**Article title:** Statistical optimization of crude oil bioremediation using *Streptomyces aurantiogriseus* isolated from Egypt's Western Desert

**Journal title:** Biodegradation

**Authors:** Sahar Y. Ibrahim^a,^*, Eman A. Abdelhamid^a,^*, Ali M. El-Hagrassi^b^, Noha M. Kamal^a,^*

**Affiliations:**

*^a^ Botany Department, Faculty of Women for Arts, Science, and Education, Ain Shams University, Cairo, Egypt. Postal code 11757*

*^b^ Department of Phytochemistry and Plant Systematics, Pharmaceutical Industries Research Institute, National Research Centre, 33 EL Buhouth St., Dokki, Giza 12622, Egypt*

***Corresponding authors:** Sahar Y. Ibrahim (E-mail: [sahar_moussa@women.asu.edu.eg](mailto:sahar_moussa@women.asu.edu.eg))

Noha M. Kamal (E-mail: [noha.ghanem@women.asu.edu.eg](mailto:noha.ghanem@women.asu.edu.eg))

Eman A. Abdelhamid (E-mail: eman.ahmed@women.asu.edu.eg)

**Supplementary Figures**


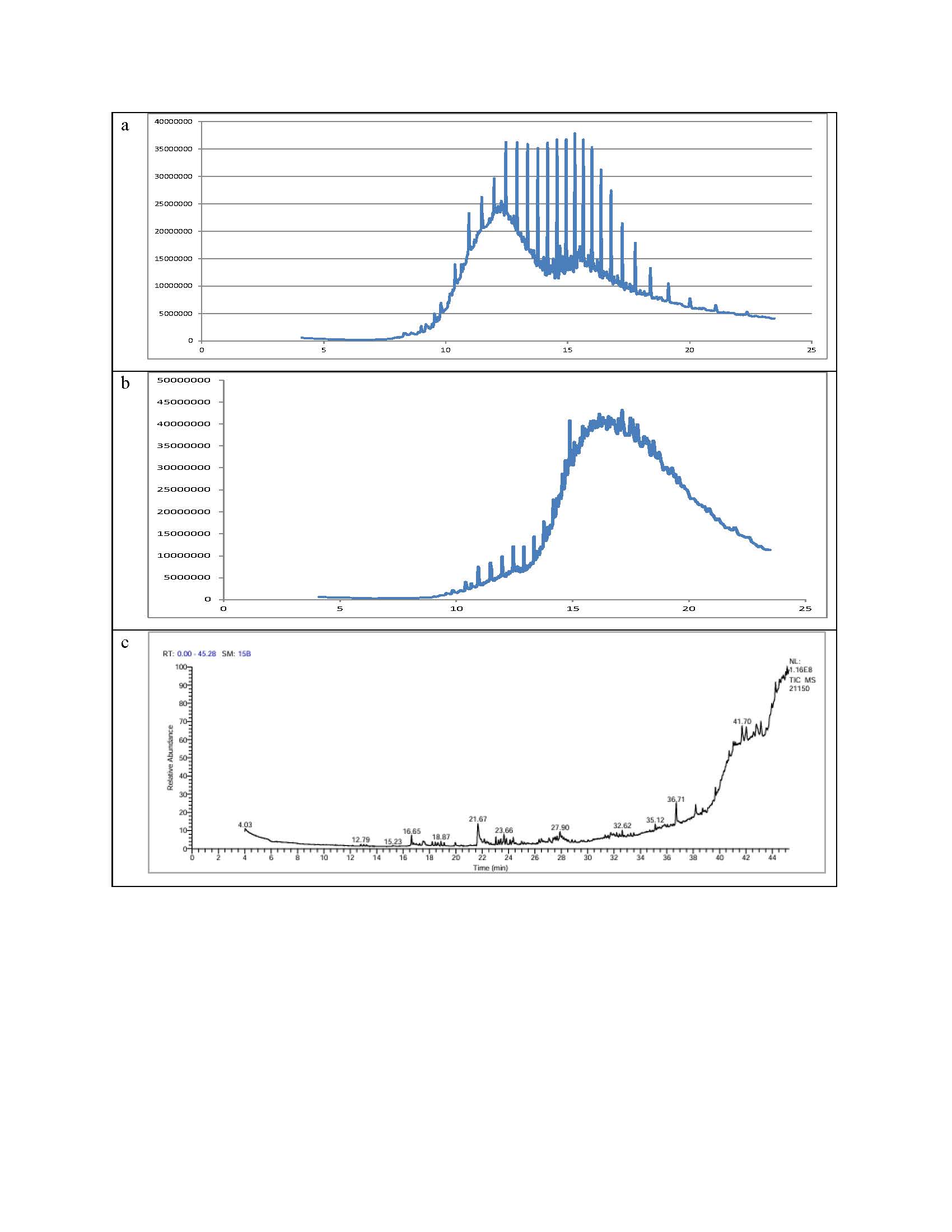


**Figure S1** GC-MS chromatograms of the polluted soil samples. a) S-CRUDE, b) S-MOTOR, and c) S-PEST. Each peak in the chromatogram corresponds to a specific compound present in the sample and peak area indicates the concentration of that compound


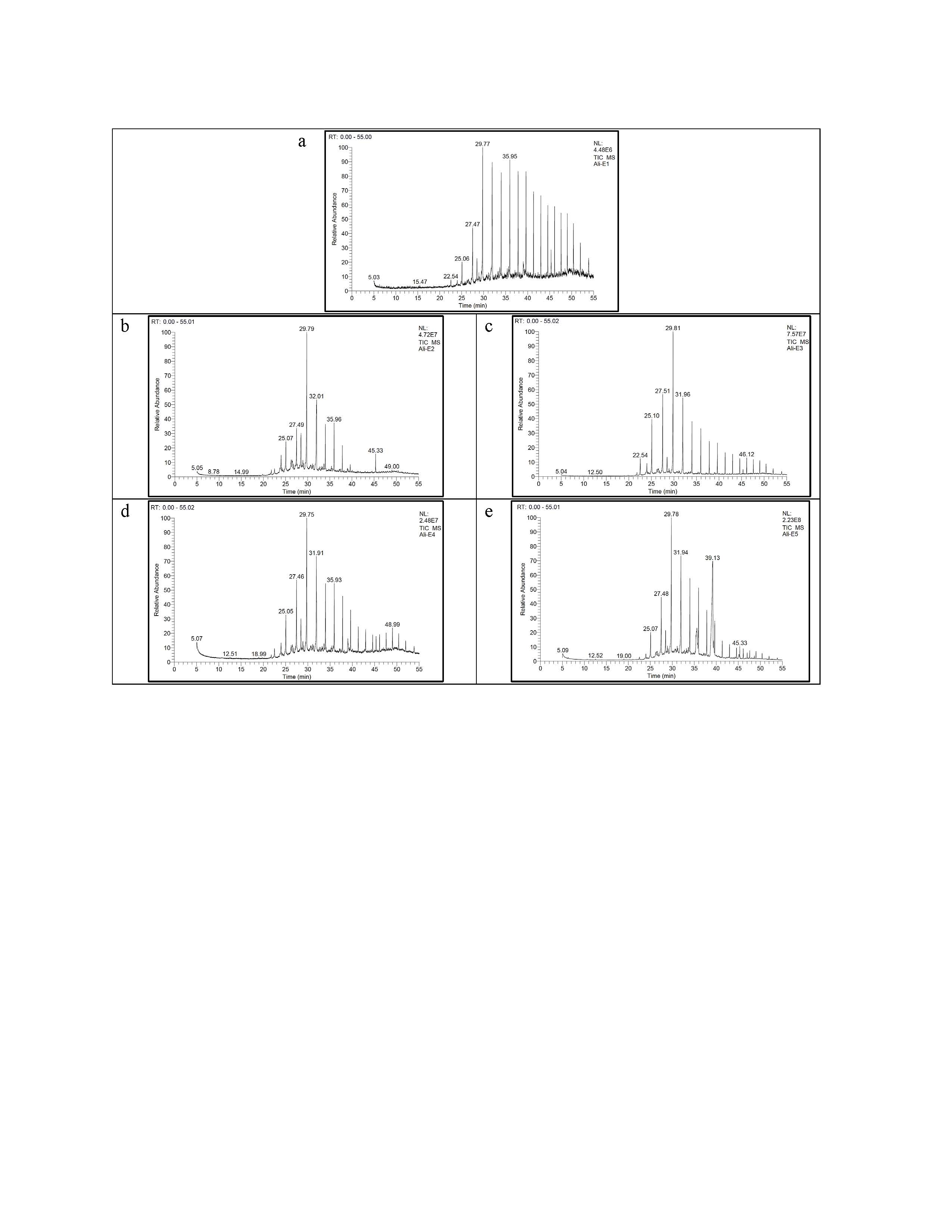


**Figure S2** GC-MS chromatograms of residual crude oil. (a) Untreated crude oil extracted using n-hexane after incubation at 30 °C and 120 rpm for 1 week without inoculation (negative control), showing higher presence of long-chain hydrocarbons, and (b, c, d, and e) treated crude oil extracted using n-hexane after treatment with isolates A2, A7, A12, and B1, respectively, and incubation at 30 °C and 120 rpm for 1 week, showing decrease in long-chain hydrocarbons


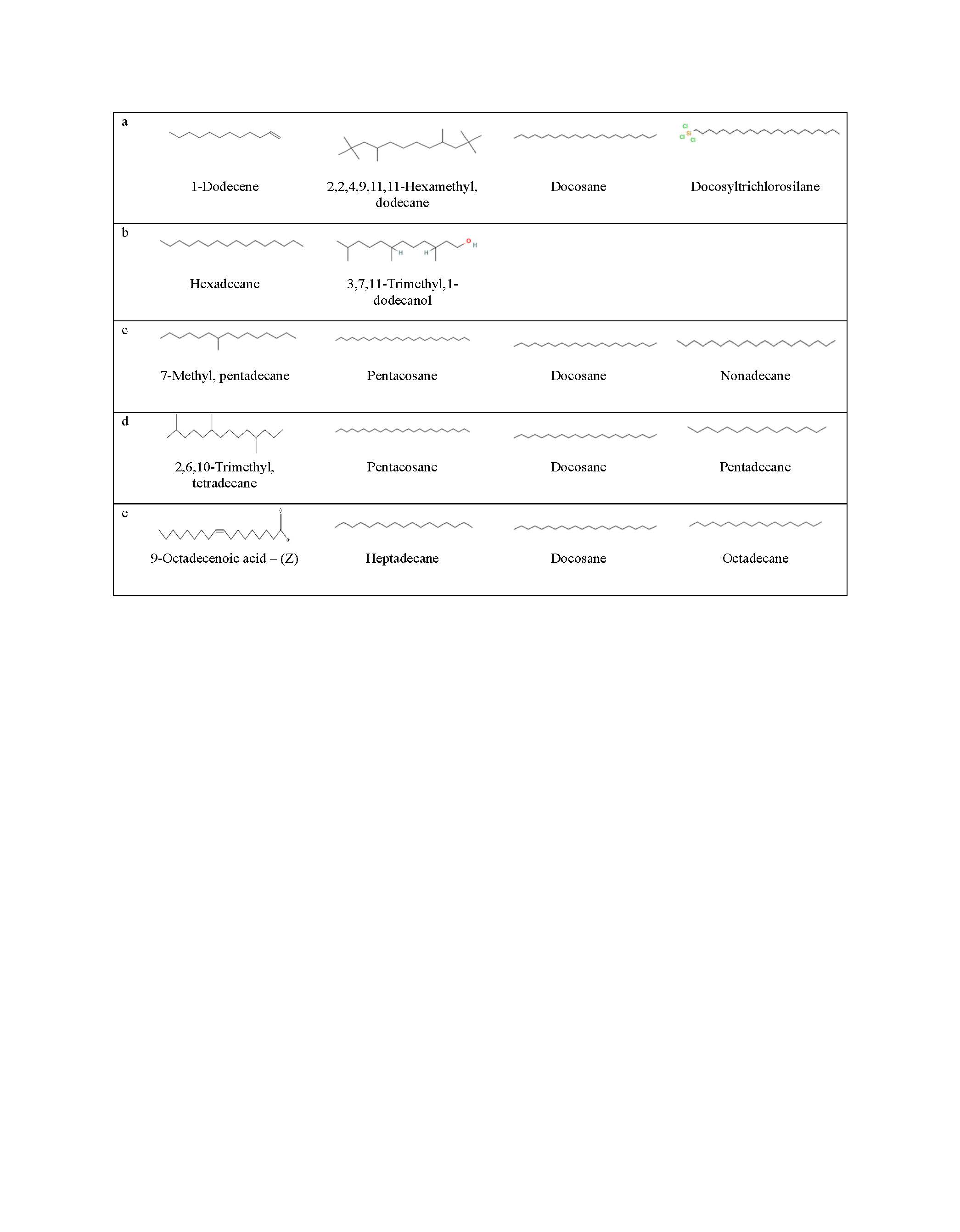


**Figure S3** Structure of the major compounds detected in GC-MS spectra. (a) Untreated crude oil (negative control), (b, c, d, and e) residual crude oil that was treated with isolates A2, A7, A12, and B1, respectively


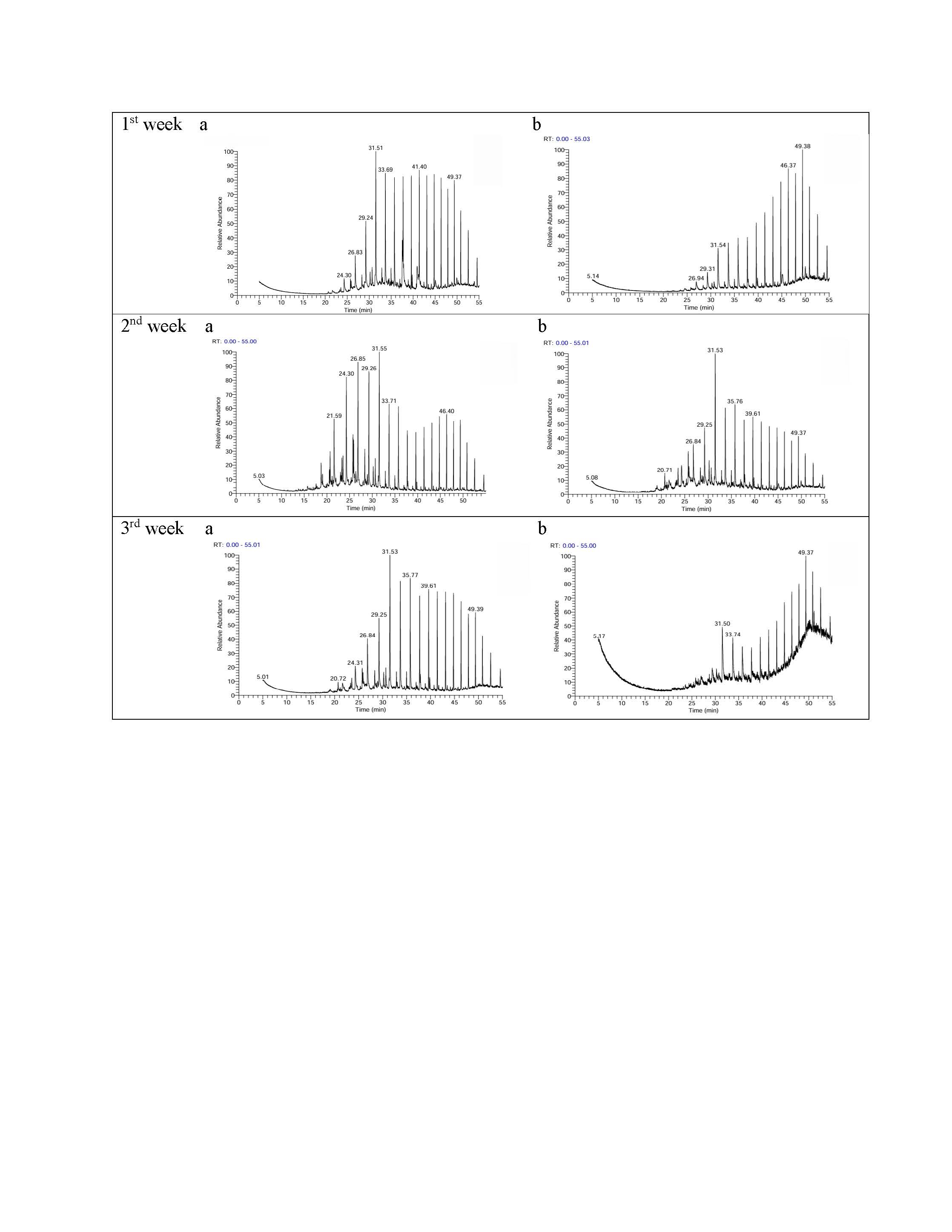


**Figure S4** GC-MS chromatograms of soil samples in the bioaugmentation pot assay. (a) Untreated soil (negative control), and (b) soil treated with isolate A7. Samples were taken and analyzed after the 1^st^, 2^nd^ and 3^rd^ weeks of incubation. Chromatograms showing a decline in long-chain alkanes (e.g., nonacosane) over time and altered composition of hydrocarbons in treated soil compared with untreated soil


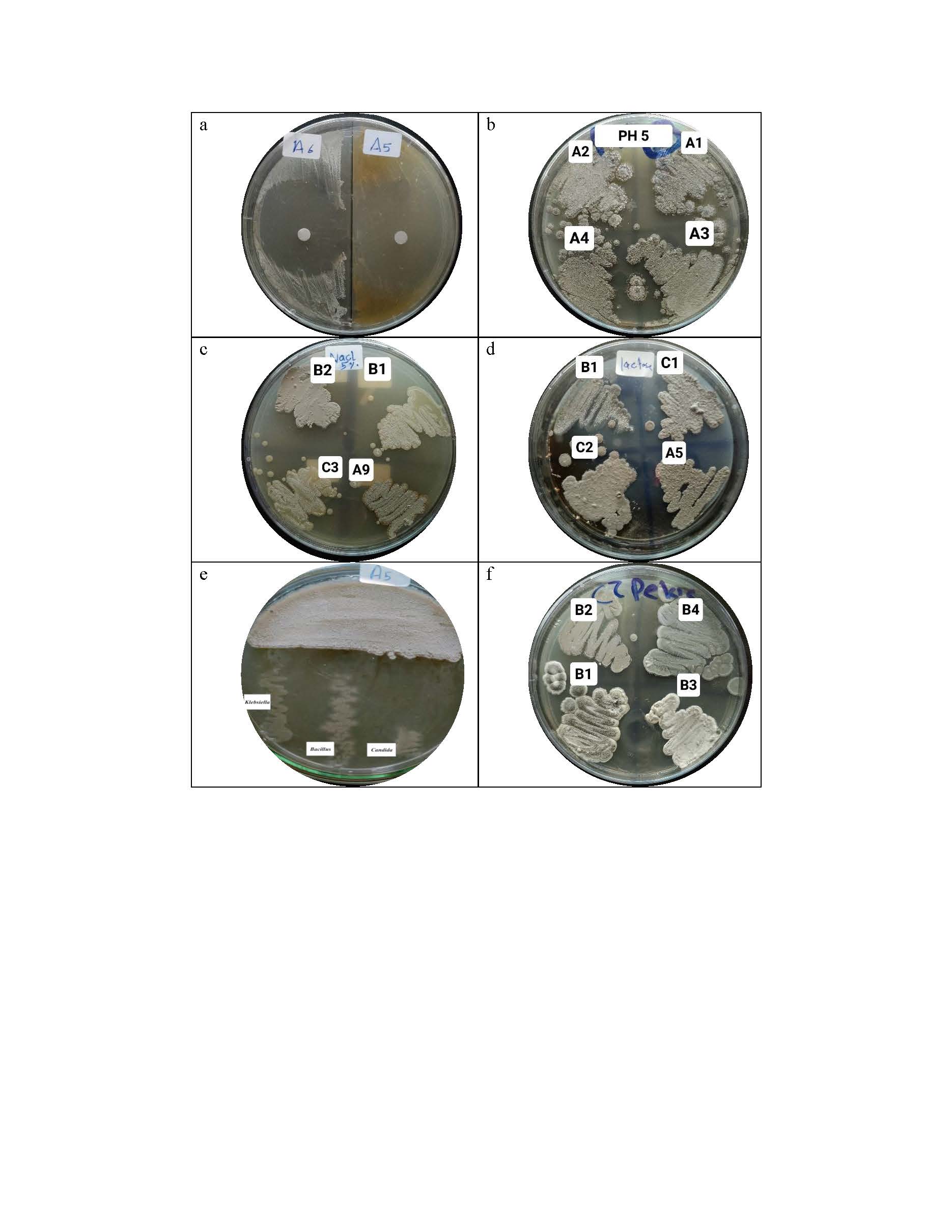


**Figure S5 S**ome of the performed tests for the phenotypic identification of the tested actinobacteria. (a) Sensitivity to streptomycin, (b) growth at pH 5, (c) growth at 5% NaCl, (d) utilization of lactose as a C source, (e) antimicrobial activity of isolate A5 against Klebsiella pneumonia, Bacillus subtilis and Candida albicans, and (f) growth on Czapek’s agar


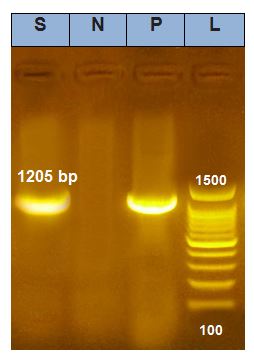


**Figure S6** Agarose gel electrophoresis of DNA of isolate A7. Lane S shows the purified DNA sample (1205 bp), N is negative control, P is positive control, and L is DNA ladder (molecular weight markers)
